# Supplementary material for: Couples data from north-western Tanzania: Insights from a survey of male partners of women enrolled in the MAISHA cluster randomized trial of an intimate partner violence prevention intervention
Source: PLoS One. 2020 Oct 2;15(10):e0240112. doi: 10.1371/journal.pone.0240112 (PMC7531846; doi:10.1371/journal.pone.0240112)
Supplement: S2 Questionnaire — (PDF) [file pone.0240112.s002.pdf]

DODOSO LA WASHIRIKI WA KIKE

DODOSO HILI LITUNZWE KWA USIRI BAADA YA KUJAZWA

SEHEMU YA 1: KUHUSU KAYA YAKO 4

SEHEMU YA 2: KUHUSU WEWE NA MWENZI WAKO 5

SEHEMU YA 3: KUHUSU WEWE NA KIPATO CHAKO 8

SEHEMU YA 4: KUHUSU WEWE NA AFYA YAKO 12

SEHEMU YA 5: KUHUSU MITIZAMO NA MILA/DESTURI ZA KIJAMII 15

SEHEMU YA 6: KUHUSU UHUSIANO WAKO 17

SEHEMU YA 7: KUHUSU KIPINDI CHA UTOTO WAKO 24

SEHEMU YA 8: KUHUSU JAMII YAKO 25

SEHEMU YA 9: WAKATI WA UFUATILIAJI TU 27

MAISHA: Utafiti wa uhusiano na Afya
Dodoso la wanawake

**Utangulizi**
Habari, jina langu ni_____________________, Ninatokea katika Taasisi ya taifa ya utafiti wa magonjwa ya binadamu (NIMR) kituo cha Mwanza na kitengo cha tafiti za kuzuia magonjwa ya binadamu (MITU) cha Mwanza. Kama unavyofahamu, wewe umekubali kushiriki katika ufafiti ambao tunaufanya kwa sasa hapa Mwanza. Miaka miwili iliyopita tulikuuliza maswali kadhaa kukuhusu wewe binafsi, kaya yako, mahusiano yako na jamii yako. Sasa tungependa kukuuliza tena baadhi ya maswali ambayo yanafanana. Baadhi ya maswali ni nyeti, lakini kila kitu utakachotuambia kitatunzwa kwa usiri na hawatashirikishwa watu wengine. Ninapenda kukusisitiza uwe mkweli, maana hakuna majibu yaliyo sahihi au yasiyokuwa sahihi. Unaweza kukatisha mahojiano wakati wowote, au unaweza ukaamua kutokujibu swali. Hii haitaathiri ushiriki wako katika utafiti huu hapo baadaye. Kama utagundua kitu chochote kinacholeta maudhi kati ya mambo tutakayojadili, na ungependa kumweleza mtu mwingine hapo baadaye, tunaweza kukusaidia kwa hili.
Mahojiano haya yatachukua masaa mawili au zaidi kumalizika. Kwa hili, itakuwa bora zaidi kama tutakuwa sehemu ambayo mahojiano yetu hayatakatishwa na ambayo haina usumbufu. Je, hapa ni mahali pazuri kufanyia mazungumzo au tunaweza kwenda mahali pengine ambapo tunaweza kuongea kwa faragha?
Je, una maswali yoyote?

KABLA YA KUANZA MAHOJIANO

**Utambulisho**	
Jina la Tawi	
1. Namba ya Tawi	[ ][ ][ ][ ]
**2.** Namba ya kikundi	[ ][ ][ ][ ]
3. Namba ya mshiriki	[ ][ ][ ]
**4.** Utambulisho wa Mshiriki	[ ][ ][ ][ ]-[ ][ ][ ][ ]-[ ][ ][ ]
**5. Je, mshiriki alihojiwa wakati wa mahojiano ya awali (Baseline)**	**1 Ndiyo 2 Hapana**<br>**Kama hapana ruka Swali namba 6**
**6. Tarehe ya mahojiano ya awali (Baseline)**	**Siku[ ][ ] /Mwezi[ ][ ][ ]/Mwaka [ ][ ][ ][ ]**<br>**Kama hakuhojiwa wakati wa baseline andika 99 kwa siku, 999 kwa mwezi na 9999 kwa mwaka**

Taarifa za Mahojiano – MWANZO

Tarehe ya mahojiano: **Siku[ ][ ] /Mwezi[ ][ ][ ]/ Mwaka [ ][ ][ ][ ]**

Muda wa kuanza mahojiano: **[ ][ ]: [ ][ ]**

Jina la mhojaji: **[ ][ ][ ]**

SEHEMU YA 1: KUHUSU KAYA YAKO

Ningependa kuanza kwa kukuuliza maswali machache kuhusu kaya yako. Ninapozungumzia kaya yako, ninamaanisha mahali na watu ambao kwa kawaida huwa mnakula chakula pamoja na mnalala katika nyumba moja.

HAKIKISHA KUWA MASWALI YOTE YANAHUSU KAYA YAKE NA SIO MAHALI MNAPOFANYIA MAHOJIANO.

	MASWALI	CODING CATEGORIES	
100	Je, nyumba unayoishi ni ya kupanga, unaimiliki (wewe mwenyewe au kwa ushirikiano na mtu mwingine) au inamilikiwa na mtu mwingine katika familia yako?	Ya kupanga	1
		Anaimiliki mwenyewe	2
		Inamilikiwa na mtu mwingine katika familia	3
		Inamilikiwa na mtu mwingine ambaye siyo sehemu ya familia yake	4
		Anamiliki kwa ushirikiano na mtu mwingine	5
101	Kaya yako inatumia vyumba vingapi kwa kulala?	[ ][ ]	
102	Ni choo cha aina gani kinatumika hasa katika kaya yako?	Vichakani	1
		Choo cha shimo ambacho hakijajengewa ukuta	2
		Choo cha kuchangia cha shimo kilichojengewa ukuta	3
		Choo binafsi cha shimo kilichojengewa ukuta	4
		Choo cha kuchangia cha shimo kilichojengewa ukuta chenye bomba la kutolea hewa chafu nje	5
		Choo binafsi cha shimo kilichojengewa ukuta na paa chenye bomba la kutolea hewa chafu nje	6
		Choo cha kuchangia cha kuflush maji	7
		Choo binafsi cha kuflush maji	8
		Sehemu nyingine (Taja)...................................	9
103	Chanzo kikuu cha mwanga katika kaya yako ni kipi hasa?	Umeme	1
		Mafuta ya taa au taa ya gesi	2
		Kuni	3
		Mshumaa	4
		Kingine (Taja)...................................	5

SEHEMU YA 2: KUHUSU WEWE NA MWENZI WAKO

Ningependa sasa kukuuliza maswali kadhaa kuhusu wewe mwenyewe na mwenzi wako.

Maswali machache yafuatayo yanamhusu mwenzi wako wa sasa au wa hivi karibuni. Tafadhali kumbuka kwamba kila kitu utakachokisema kitatunzwa kwa usiri na hakitaelezwa kwa mtu yeyote kutoka katika jamii yako.

	MASWALI	CODING CATEGORIES	NENDA HADI
200.	Kwa sasa umeolewa au unaishi na mwanaume kama vile mmeoana?	Ndiyo 1<br>Hapana 2	202
201.	Umekuwa kwenye mahusiano ya kimapenzi na mwanaume yeyote ndani ya miezi 12 iliyopita?	Ndiyo 1<br>Hapana 2	 215
202.	Je, huyu mwanaume ni yule yule uliyekuwa naye miaka miwili iliyopita wakati unafanyiwa mahojiano ya awali (au ya mara ya kwanza) mwanzoni mwa utafiti huu? Mahojiano ya awali yalifanyika [SHOW DATE SHE WAS INTERVIEWED]	Ndiyo 1<br>Hapana 2	215
	Kama mwenzi ni tofauti uliza swali 203 hadi 214 Maswali yafuatayo yanamhusu mwanaume ambaye unamchukulia kama mume au mwenzi wako wa kudumu.		
203.	Kwa muda gani umekuwa (ulikuwa) katika uhusiano huu?<br>MSHIRIKI AKADIRIE KAMA HAFAHAMU MUDA HALISI. KAMA HAFAHAMU MWEZI WEKA 00	Miaka: [ ][ ] Miezi: [ ][ ]	
204.	Mwenzi wako ameoa (alikuwa ameoa) mwanamke mwingine au ana mpenzi mwingine?	Ndiyo 1<br>Hapana 2<br>Sijui 3	 206<br>206
205.	Wewe ni (ulikuwa) mke/mpenzi wa kwanza, wa pili, ...mke wa ngapi?<br>JAZA 96 KAMA HAFAHAMU	Mke wa ngapi: [ ][ ]	
206.	Mwenzi wako ana (alikuwa na) umri gani? JAZA 96 KAMA HAFAHAMU	Miaka: [ ][ ]	
207.	Mwenzi wako ni (alikuwa) dini gani?<br>ZUNGUSHIA JIBU MOJA	Muislam 1<br>Msabato 2<br>Tanzania Assemblies of God (TAG) 3<br>Mkatoliki 4<br>Mlutheri 5<br>Moravian 6<br>Pentekoste 7<br>African Inland Church (AIC) 8<br>Sijui 9<br>Nyingine: 10	

208.	Mwenzi wako ni (alikuwa) kabila gani?<br><br>ZUNGUSHIA JIBU MOJA	Msukuma<br>Mjita<br>Mzinza<br>Mnyiramba<br>Mkara/Mkerewe<br>Mhaya<br>Mjaluo<br>Mkuria/Mshashi<br>Mchaga<br>Mhindi<br>Mwarabu<br>Sijui<br>Nyingine	1<br>2<br>3<br>4<br>5<br>6<br>7<br>8<br>9<br>10<br>11<br>12<br>13	
209.	(Alikuwa) amemaliza kiwango gani cha juu cha elimu?<br><br>ZUNGUSHIA JIBU MOJA	Hajaenda shule kabisa<br>Hajamaliza shule ya msingi<br>Amemaliza shule ya msingi<br>Hajamaliza shule ya sekondari<br>Sekondari (Kidato cha 1-IV)<br>Sekondari (Kidato cha V-VI)<br>Mafunzo ya chuo mara baada ya shule ya msingi/sekondari na kabla ya chuo kikuu<br>Chuo kikuu<br>Sijui	1<br>2<br>3<br>4<br>5<br>6<br>7<br>8<br>9	
210.	Mwenzi wako amekuwa akifanya kazi kwa ajili ya kupata pesa ndani ya miezi 12 iliyopita? (hii inaweza kuwa kwa kuajiriwa au yeye binafsi kujiajiri)	Ndiyo<br>Hapana	1<br>2	 215
211.	Kazi hii ni (ilikuwa) ajira binafsi au ameajiriwa (aliajiriwa)?	Ajira binafsi<br>Aliajiriwa	1<br>2	
212.	Je, mwenzi wako huwa mara kwa mara anafanya kazi kwa mwaka mzima, anafanya kazi kwa msimu, au mara mojamoja?	Mwaka mzima<br>Kwa msimu/kipindi fulani katika mwaka<br>Mara mojamoja	1<br>2<br>3	
213.	Je, mwenzi wako huwa anasafiri mbali na nyumbani kwa ajili ya kazi yake? Unaweza kusema ni kwa muda gani katika miezi 12 iliyopita:<br><br>SOMA ORODHA YA MAJIBU<br>ZUNGUSHIA JIBU MOJA	Zaidi ya nusu mwaka<br>Kama nusu mwaka<br>Chini ya nusu mwaka<br>Hasafiri kabisa	1<br>2<br>3<br>4	

214.	Ndani ya miezi 12 iliyopita, kwa kawaida mwenzi wako alipata kipato cha pesa kiasi gani, kwa mwezi au kwa wiki?<br><br>WEKA 969696 KAMA HAFAHAMU	Shilingi za kitanzania kwa mwezi (kadiria)<br>:[ ][ ] [ ][ ] [ ][ ]<br><br>Shilingi za kitanzania kwa wiki (kadiria)<br>[ ][ ] [ ][ ] [ ][ ]	

Ningependa kujifunza zaidi juu ya watoto ambao unawajibika nao, hii inajumuisha watoto wako mwenyewe na watoto wengine ambao unaishi nao, pamoja na watoto wanaoishi mahali pengine.Tafadhali kumbuka taarifa hii ni ya siri. Tunapotumia herufi za mwanzo za majina ya watoto wako ni kwa ajili tu ya kusaidia kutambua ni mtoto yupi ndiye tunayemzungumzia. Hii haiwezi kutumika kumtambulisha mtoto wako kwa watu wengine.

TUMIA SEHEMU HII YA MAHOJIANO KUJENGA UKARIBU NA MSHIRIKI

Una watoto wenye umri usiozidi miaka 18 ambao unawajibika nao, ikijumuisha wale wanaoishi katika kaya yako na wale wanaoishi mahali pengine?	Ndiyo<br>Hapana	1<br>2	Kama jibu ni hapana nenda swali la 300

**215**<br>**216**	Ni watoto wangapi wenye umri usiozidi miaka 18 ambao unawajibika nao, ikijumuisha wale wanaoishi katika kaya yako na wale wanaoishi mahali pengine?	215.<br>Idadi ya unaoishi nao<br><br>[ ][ ]	216.<br>Idadi ya wanaoishi mahali pengine<br><br>[ ][ ]

217-222	Nitakuuliza kidogo kuhusu kila mtoto, kuanzia yule mkubwa zaidi.	ANZA NA YULE MKUBWA ZAIDI.<br>Idadi ya watoto (1-6)	217.	218.	219.	220.	221.	222.
a.	Herufi za kwanza za majina yao au majina ya utani ni zipi?	JAZA HERUFI **ZOTE** ZA KWANZA ZA MAJINA YAO MATATU						
b.	Je, huyu ni mvulana au msichana?	Kiume<br><br>Kike	1<br><br>2	1<br><br>2	1<br><br>2	1<br><br>2	1<br><br>2	1<br><br>2
c.	Alizaliwa tarehe ngapi?	Siku<br><br>Mwezi (MMM)<br><br>Mwaka<br><br>(TUMIA 96 KWA WOTE KAMA HAIFAHAMIKI)	[ ][ ]<br><br>_ _ _<br><br>[] [] [] [ ]	[ ][ ]<br><br>_ _ _<br><br>[] [] [] [ ]	[ ][ ]<br><br>_ _ _<br><br>[] [] [] [ ]	[ ][ ]<br><br>_ _ _<br><br>[] [] [] [ ]	[ ][ ]<br><br>_ _ _<br><br>[] [ ] [] []	[ ][ ]<br><br>_ _ _<br><br>[] [] [] []
d.	Ana umri gani?	KAMA TAREHE HAIFAHAMIKI, WEKA 96<br>KAMA CHINI YA MWAKA 1, WEKA 01	[ ][ ]	[ ][ ]	[ ][ ]	[ ][ ]	[ ][ ]	[ ][ ]
e.	Je, [JINA] anaishi na wewe?	Hapana, anaishi kaya nyingine<br><br>Hapana, yuko shule<br><br>Ndiyo	1<br><br>2<br><br>3	1<br><br>2<br><br>3	1<br><br>2<br><br>3	1<br><br>2<br><br>3	1<br><br>2<br><br>3	1<br><br>2<br><br>3

f.	Wewe ni mama yake?	Ndiyo<br>Hapana-mwanamke mwingine	1<br>2	1<br>2	1<br>2	1<br>2	1<br>2	1<br>2
g.	Baba yake ni mwenzi wako wa sasa au mwanaume mwingine?	Mwenzi wa sasa<br>Mwenzi wa zamani<br>Mwanaume mwingine	1<br>2<br>3	1<br>2<br>3	1<br>2<br>3	1<br>2<br>3	1<br>2<br>3	1<br>2<br>3
h.	Je [JINA] yuko shule?	Hajawahi kusoma (RUKA HADI SWALI LA J)<br>Zamani<br>Sasa hivi	1<br>2<br>3	1<br>2<br>3	1<br>2<br>3	1<br>2<br>3	1<br>2<br>3	1<br>2<br>3
i.	Amefikia kiwango gani cha juu cha elimu	Shule ya msingi<br>Shule ya sekondari<br>Shule ya nursery<br>Chuo	1<br>2<br>3<br>4	1<br>2<br>3<br>4	1<br>2<br>3<br>4	1<br>2<br>3<br>4	1<br>2<br>3<br>4	1<br>2<br>3<br>4
j.	KAMA HAJAANDIKISHWA SHULE: Nini sababu <u>kubwa</u> iliyopelekea asiwe shule [KUMBUKA: usisome majibu]	Utoro<br>Ujauzito<br>Kuolewa<br>Karo ya shule<br>Ukosefu wa pesa<br>Mgonjwa<br>Kutunza familia<br>Kufeli mitihani<br>Kulima/kazi za nyumbani<br>Mdogo sana<br>Nyingine	1<br>2<br>3<br>4<br>5<br>6<br>7<br>8<br>9<br>10<br>11<br>12	1<br>2<br>3<br>4<br>5<br>6<br>7<br>8<br>9<br>10<br>11<br>12	1<br>2<br>3<br>4<br>5<br>6<br>7<br>8<br>9<br>10<br>11<br>12	1<br>2<br>3<br>4<br>5<br>6<br>7<br>8<br>9<br>10<br>11<br>12	1<br>2<br>3<br>4<br>5<br>6<br>7<br>8<br>9<br>10<br>11<br>12	1<br>2<br>3<br>4<br>5<br>6<br>7<br>8<br>9<br>10<br>11<br>12
k.	KAMA AMEACHA SHULE:, Aliacha akiwa na umri gani?		[ ][ ]	[ ][ ]	[ ][ ]	[ ][ ]	[ ][ ]	[ ][ ]
l.	KAMA BADO YUKO SHULE: kwa sasa anarudia mwaka?	Ndiyo<br>Hapana	1<br>2	1<br>2	1<br>2	1<br>2	1<br>2	1<br>2

SEHEMU YA 3: KUHUSU WEWE NA KIPATO CHAKO

Sasa ningependa kufahamu zaidi juu ya njia unazozitumia kujipatia pesa.

300.	Je, chanzo kikuu cha mapato yako na familia yako ni kipi?<br><br>INARUHUSU MAJIBU ZAIDI YA MOJA	Ndiyo	Hapana	
**a.**	Pesa kutokana na shughuli binafsi	1	2	
**b.**	Msaada kutoka kwa mume/mwenzi	1	2	
**c.**	Msaada kutoka kwa ndugu wengine	1	2	
**d.**	Malipo baada ya kustaafu	1	2	
**e.**	Huduma/ustawi wa jamii	1	2	
**f.**	Nyingine (Taja)	…………………………		
301.	Je, umewahi kujipatia pesa yako mwenyewe katika kipindi cha miezi 12 iliyopita?	Ndiyo 1<br><br>Hapana 2		 <br>320
302.	Je, umejiajiri wewe mwenyewe au umeajiriwa na mtu mwingine/shirika?	Nimejiajiri 1<br><br>Nimeajiriwa na mtu mwingine au shirika 2<br><br>Yote ni kweli 3		310
303.	(KAMA AMEAJIRIWA NA MTU MWINGINE AU SHIRIKA),<br>Mwajiri wako ni nani hasa?	Ndugu 1<br><br>Jirani 2<br><br>Rafiki/mtu anayefahamiana naye 3<br><br>Serikali 4<br><br>Shirika lisilo la kiserikali 5<br><br>Kampuni/biashara ya mtu binafsi 6<br><br>Mwingine (Taja): 7		

Maswali yafuatayo yanahusu kazi unayoifanya (uliyoifanya) wakati umeajiriwa na mtu au shirika
Ruka sehemu hii kama hajaajiriwa na mtu mwingine au shirika.

304.	Je, kazi hii ni ya kudumu au unaifanya mara mojamoja?	Kudumu 1<br><br>Mara mojamoja 2	
305.	Je, kwa kawaida huwa unafanya kazi mwaka mzima, au unafanya kwa msimu, au mara mojamoja?	Mwaka mzima 1<br><br>Msimu/kipindi fulani cha mwaka 2<br><br>Mara moja moja 3	
306.	Kazi hii umeifanya kwa jumla ya miezi mingapi ndani ya miezi 12 iliyopita?<br>Unaweza kusema...<br><br>SOMA MAJIBU	Mwezi 1 au chini ya mwezi 1 1<br><br>Miezi 1 - 6 2<br><br>Miezi 6 - 9 3<br><br>Miezi 9 - 12 4	

307.	Unalipwa malipo yako kwa pesa taslimu, kupewa chakula au haulipwi kabisa?	Pesa taslimu tu	1	
		Pesa taslimu na vitu	2	
		Ninapewa vitu pekee	3	309
		Nyingine	4	309
		Silipwi kabisa	5	309
308.	Kwa kawaida unapata pesa kiasi gani kutokana na kazi hii, kwa siku au wiki au mwezi?<br><br>JAZA MOJA TU; KAMA SIKU, WIKI AU MWEZI. JAZA 969696 KAMA HAJUI	Siku:	[ ] [ ] [] [ ] [] [ ]	
		Wiki:	[ ] [ ] [] [ ] [] [ ]	
		Mwezi:	[ ] [ ] [] [ ] [] [ ]	
309.	Je, una mpango wa kuendelea kufanya kazi hii ?	Ndiyo	1	
		Hapana	2	
		Sijui	96	
	Kama amejiajiri…			
310.	Huwa unafanya biashara ya aina gani?<br><br>(NI SAWA KUKIWA NA MAJIBU MENGI)		Ndiyo / Hapana	
		a. Kuuza mboga	1 / 2	
		b. Kuuza vyakula vilivyotengenezewa nyumbani	1 / 2	
		c. Kuendesha mgahawa mdogo	1 / 2	
		d. Kushona au kutengeneza nguo	1 / 2	
		e. Kuuza nguo	1 / 2	
		f. Kutengeneza/kuuza pombe	1 / 2	
		g. Kuendesha saluni	1 / 2	
		h. Kuendesha duka	1 / 2	
		i.kuuza samaki	1 / 2	
		j. Nyingine (Taja)…………………	1 / 2	
311.	Kuhusiana na biashara yako kuu, umekuwa ukiendesha biashara hii kwa zaidi au chini ya miezi 12?	Chini ya miezi 12	1	
		Zaidi ya miezi 12	2	
312.	Je, wewe ndiye unayewajibika peke yako katika shughuli hii au kuna watu wengine wanaowajibika?	Mimi ndiye ninayewajibika hasa	1	
		Wengine wanawajibika	2	
313.	Ndani ya miezi 12 iliyopita, ni watu wengine wangapi katika kaya yako ambao wamechangia muda, pesa au mawazo katika kazi hii ?<br><br>INGIZA 96 KAMA HAJUI	Idadi:	[ ] [ ]	

314.	Umefanya kazi hii kwa siku ngapi katika mwezi uliopita?<br><br>INGIZA 96 KAMA HAJUI	Idadi: [ ] [ ]		
315.	Kwa kawaida huwa unafanya kazi kwa masaa mangapi kwa siku,?<br><br>INGIZA 96 KAMA HAJUI	Saa: [ ] [ ]		
316.	Katika siku /wiki/mwezi wa kawaida, huwa unapata kipato kiasi gani kutokana na kazi hii?<br><br>JAZA SIKU, WIKI AU MWEZI	Siku: [ ] [ ] [] [ ] [] [ ]<br>wiki: [ ] [ ] [] [ ] [] [ ]<br>Mwezi: [ ] [ ] [] [ ] [] [ ]		
317.	Katika kipindi cha mwezi mmoja uliyopita, ni mara ngapi hukupata kiasi cha pesa cha kutosheleza gharama za kuendesha biashara yako?	Haijawahi kutokea<br>Mara moja<br>Mara chache<br>Mara nyingi	1<br>2<br>3<br>4	
318.	Je, umeajiri watu wengine na unawalipa mshahara?	Ndiyo<br>Hapana	1<br>2	
319.	Katika kiasi cha pesa ulichopata ndani ya mwezi mmoja uliyopita, uliwekeza kiasi gani ili kupanua biashara yako?	Sikuwekeza kiasi chochote<br>Kiasi kidogo<br>Nusu yake<br>Kiasi kikubwa<br>Kiasi chote	1<br>2<br>3<br>4<br>5	

	MASWALI	CODING CATEGO RIES			NENDA
	Ningependa sasa nikuulize maswali kuhusu kama umekuwa ukitumia asasi inayotoa mikopo midogo midogo ya kifedha, kama vile SACCOS, BRAC, FINCA au mashirika mengine?				
320.	Ndani ya miezi 12 iliyopita, je, umechukua mkopo wa fedha kutoka kwenye mashirika/asasi yoyote inayotoa mikopo midogomidogo tofauti na BRAC? Kama vile SACCOS,, FINCA, PRIDE au asasi nyingine?		Ndiyo<br>Hapana	1<br>2	 324
321.	Umekopa pesa kutoka kwenye asasi gani ndani ya miezi 12 iliyopita tofauti na BRAC?	a. FINCA<br>b. SACCOS<br>c. PRIDE<br>d. JIJENGE<br>e.Nyingine (Taja)…………….	Ndiyo<br>1<br>1<br>1<br>1<br>1	Hapana<br>2<br>2<br>2<br>2<br>2	
322.	Mpaka sasa bado unakopa pesa kutoka katika moja wapo ya asasi/mashirika haya?		Ndiyo<br>Hapana	1<br>2	

323.	Kuhusu mkopo wako wa sasa ambao **haukukopa BRAC**, ulikopa pesa kiasi gani?<br><br>WEKA 0 KWENYE SEHEMU ZOTE ZA KUANDIKA KAMA KWA SASA AMEKOPA PESA BRAC TU,NA SIO KWENYE ASASI/ MASHIRIKA MENGINE	Kiasi [ ] [ ] [ ] [ ] [ ] [ ] [ ]	
324.	Kwa sasa una mkopo kutoka BRAC?	Ndiyo 1<br>Hapana 2	 332
325.	Kuhusu mkopo wako wa hivi sasa uliopata kutoka BRAC, ulikopa pesa kiasi gani? (TUMIA 96 KAMA HAJUI)	Kiasi [ ] [ ] [ ] [ ] [ ] [ ] [ ]	
326.	Ndani ya miezi 12 iliyopita, je, umekuwa ukikopa pesa kutoka BRAC kwa mfululizo au kuna wakati ulikatisha kukopa pesa kutoka kwao?	Mfululizo 1<br>Nilikatisha 2<br>Mkopo mpya 3	328<br><br>328
327.	Kwa nini ulisitisha kukopa pesa kutoka BRAC?	Nilikuwa sihitaji tena 1<br>Sikuweza kurejesha mkopo 2<br>Mume alikataa kuchukua mkopo mpya 3<br>Nyingine (Taja)…………………………………. 4	
328.	Je, pesa uliyokopa uliitumia kwa sababu gani ya **msingi**?<br><br>MSOMEE MAJIBU	Kuanzisha biashara ndogo 1<br>Kuendeleza biashara 2<br>Kulipia gharama za matibabu 3<br>Kulipia gharama za shule 4<br>Kununulia chakula au nguo 5<br>Kurejesha mkopo mwingine 6<br>Kulipia kodi ya nyumba 7<br>Kusaidia watu wengine katika familia 8<br>Nyingine (Taja)………………………….. 9	
329.	Je, unajiamini kiasi gani kwamba utaweza kurejesha huu mkopo?<br><br>Unaweza kusema:<br>unajiamini sana<br>unajiamini kiasi<br>hujiamini	Najiamini sana 1<br>Najiamini kiasi 2<br>Sijiamini 3	
330.	Pesa uliyopata kutoka BRAC na vikundi vingine vya mikopo midogomidogo ni ya muhimu kiasi gani?<br><br>Unaweza kusema kwamba ni muhimu sana, ni muhimu kiasi, au sio muhimu kabisa.	Muhimu sana 1<br>Muhimu kiasi 2<br>Sio muhimu kabisa 3	

331.	Ndani ya miezi 12 iliyopita, kwa ujumla, ni kwa jinsi gani kuwa mwanachama wa BRAC au vikundi vingine vya mikopo midogomidogo kumeathiri uhusiano wako na mwenzi wako? Unaweza kusema kwamba: hakujaathiri uhusiano na mwenzi wako, kumefanya uhusiano wako uwe na matatizo zaidi, au kumeboresha uhusiano wako?	Hakujaathiri uhusiano<br>Kumefanya uhusiano uwe na matatizo zaidi<br>Kumeboresha uhusiano<br>Hana uhusiano	1<br>2<br>3<br>9	

	MASWALI	CODING CATEGORIES			
332.	Siku hizi, familia nyingi zina wakati mgumu katika kufikia malengo. Ningependa kujifunza zaidi jinsi familia yako inavyojimudu.<br><br>SISITIZA KWAMBA UNAZUNGUMZIA KUHUSU MIEZI 12 ILIYOPITA<br><br>Ndani ya miezi 12 iliyopita...		**Kama imewahi kutokea** tafadhali jaza jibu lake upande wenye kivuli		
		Haijawahi kutokea	Mara moja	Mara chache	Mara nyngi
a.	...ulikuwa na wasiwasi mkubwa juu ya hali yako ya kifedha kwa ujumla?<br>Imewahi kutokea au haijawahi kutokea?	1	2	3	4
b.	...umepata tatizo la kununua chakula au mahitaji mengine muhimu ya familia yako?<br>Imewahi kutokea au haijawahi kutokea?	1	2	3	4
c.	...ulilazimika kukopa pesa ili kulipa kodi ya nyumba au bili nyingine?<br>Imewahi kutokea au haijawahi kutokea?	1	2	3	4
d.	...mmojawapo wa wanafamilia alihitaji kuonana na daktari lakini hakuweza kwa sababu haukuwa na pesa ya kutosha?<br>Imewahi kutokea au haijawahi kutokea?	1	2	3	4
e.	...watoto wako walikosa kwenda shule kwa sababu haukuwa na pesa ya ada, sare za shule au mahitaji?<br>Imewahi kutokea au haijawahi kutokea?	1	2	3	4
f.	...ilitokea wewe au mtoto wako yeyote hakula kitu chochote siku nzima kwa sababu hakukuwa na chakula cha kutosha?<br>Imewahi kutokea au haijawahi kutokea?	1	2	3	4

	MASWALI	CODING CATEGORIES	
333.	Ndani ya miezi 12 iliyopita, kwa kiasi gani pesa unayoleta (uliyoleta) wewe binafsi katika familia ilikuwa ni muhimu? Unaweza kusema kwamba ni muhimu kupindukia, muhimu sana, ni muhimu kiasi, sio muhimu sana, au haleti pesa yoyote.	Muhimu kupindukia<br>Muhimu sana<br>Muhimu kiasi<br>Sio muhimu sana<br>Haleti pesa yoyote	1<br>2<br>3<br>4<br>5
334.	Unaweza kusema kwamba pesa unayoleta katika kaya ni nyingi kuliko anayochangia mume/mwenzi wako? Kidogo kuliko anayochangia, au ni karibu sawa na anayochangia?	Kidogo kuliko mwenzi<br>Sawa na mwenzi<br>Zaidi ya mwenzi<br>Hana mwenzi	1<br>2<br>3<br>4
335.	Je, unadhani kwamba unaweza kujihudumia wewe mwenyewe na familia yako kwa kutumia kipato chako pekee? Unaweza kusema ndiyo kabisa, ndiyo kwa shida, pengine haiwezekani, au haiwezekani kabisa	Ndiyo kabisa<br>Ndiyo kwa shida<br>Pengine haiwezekani<br>Haiwezekani kabisa	1<br>2<br>3<br>4

SEHEMU YA 4: KUHUSU WEWE NA AFYA YAKO

Maswali yanayofuata yanakuhusu wewe na afya yako

400	Ningependa kujifunza jinsi unavyoendesha maisha yako, kwa ujumla. Nitakusomea sentensi kadhaa, na ningependa wewe useme kama ni za kweli au sio-kweli.<br><br>KAMA KWELI,<br>ULIZA KWELI KIDOGO, KWELI AU KWELI KABISA<br><br>KAMA SIO-KWELI,<br>ULIZA SIO-KWELI KIDOGO, SIO-KWELI AU SIO-KWELI KABISA	Kweli kabisa	kweli	Kweli kidogo	Sio-kweli kidogo	Sio kweli	Sio kweli kabisa
a.	Huwezi kufikiria juu ya njia mbalimbali za kujikwamua kutoka katika hali ngumu iwapo maisha yatabadilika bila kutegemea.	1	2	3	4	5	6
b.	Unafuatilia malengo yako kwa bidii.	1	2	3	4	5	6
c.	Muda mwingi unajisikia mchovu.	1	2	3	4	5	6
d.	Kuna njia nyingi sana za kutatua tatizo lolote linalojitokeza.	1	2	3	4	5	6
e.	Wewe huwa mara nyingi unaingia katika mabishano	1	2	3	4	5	6
f.	Huwezi kufikiria njia nyingi sana za kuweza kupata vitu unavyovihitaji katika maisha ambavyo ni vya muhimu kwako	1	2	3	4	5	6
g.	Wewe huwa unakuwa na wasiwasi juu ya afya yako.	1	2	3	4	5	6
h.	Hata pale wengine wanapokata tamaa, wewe unajua unaweza kupata njia ya kutatua tatizo.	1	2	3	4	5	6
i.	Uzoefu wa zamani haujakuandaa vizuri kwa ajili ya maisha ya baadaye.	1	2	3	4	5	6
j.	Wewe umekuwa mtu mwenye mafanikio sana katika maisha.	1	2	3	4	5	6
k.	Mara kwa mara huwa unajikuta unahofia kitu fulani.	1	2	3	4	5	6
l.	Wewe huwa huyafikii malengo uliyojiwekea mwenyewe.	1	2	3	4	5	6

Sasa ningependa nikuulize maswali kadhaa kuhusu afya yako.

401	Ndani ya wiki 4 zilizopita, umesumbuliwa na mojawapo kati ya matatizo yafuatayo?	Ndiyo	Hapana
a.	Je, mara kwa mara huwa unaumwa kichwa?	1	2
b.	Je, hamu yako ya kula huwa sio nzuri?	1	2
c.	Je, huwa unalala vibaya? Kama vile kukosa usingizi, kuamka usiku wa manane zaidi ya mara tatu, au kuamka asubuhi sana kisha ukakosa usingizi?	1	2
d.	Je, huwa unapata hofu kirahisi?	1	2

e.	Je, mikono yako huwa inatetemeka?	1	2
f.	Je, huwa unasikia uoga au wasiwasi?	1	2
g.	. Je, mfumo wa kusaga chakula mwilini mwako huwa sio mzuri? Kama vile kutopata choo mara kwa mara, unasikia kichefuchefu au hauna hamu ya kula.	1	2
h.	Je, huwa unapata shida kufikiria kwa umakini?	1	2
i.	. Je, huwa unajisikia hauna furaha?	1	2
j.	Je, huwa unalia zaidi kuliko kawaida kama vile kila siku, au zaidi ya mara moja kwa siku. kwa sababu ya matatizo?	1	2
k.	Je, huwa inakuwa vigumu kwako kufurahia kazi zako za kila siku?	1	2
l.	Je, huwa unaona ni vigumu kwako kufanya maamuzi yoyote?	1	2
m.	Je, kazi yako kuu ya kila siku huwa haiendi vizuri?	1	2
n.	Je, huwa unashindwa kutoa mchango au kufanya mambo muhimu katika maisha?	1	2
o.	Huwa unapoteza hamasa ya kufanya mambo?	1	2
p.	Je, huwa unajisikia kuwa mtu asiyekuwa na thamani?	1	2
q.	Je, mawazo ya kujiua yamekuwa yakikujia akilini?	1	2
r.	Je, huwa unajisikia vibaya tumboni?	1	2
s.	Je, huwa unachoka kirahisi?	1	2
402	Katika swali linalofuata tungependa kujua ni kwa kiasi gani unajisikia kuridhika na maisha yako kwa ujumla.		
a	Kwa ujumla, umeridhika kwa kiasi gani na maisha yako siku hizi?	Siridhiki kabisa<br>Naridhika kidogo<br>Naridhika kiasi (au kwa wastani)<br>Naridhika kabisa	1<br>2<br>3<br>4
	Swali linalofuata linahusu jinsi unavyojisikia kuhusu thamani ya vitu unavyofanya katika maisha yako kwa ujumla.		
b	Kwa ujumla, vitu unavyofanya katika maisha yako unahisi vina thamani?	Havina thamani kabisa<br>Vina thamani kidogo<br>Vina thamani kiasi (au kwa wastani)<br>Vina thamani kabisa	1<br>2<br>3<br>4

	Maswali yanayofuata yanahusu jinsi ulivyojisikia jana kwa ujumla.		
c.	Je, jana ulijisikia mwenye furaha?	Sikuwa na furaha kabisa	1
		Nilikuwa na furaha kidogo	2
		Nilikuwa na furaha kiasi (kwa wastani)	3
		Nilikuwa na furaha kabisa	4
d.	Je, jana ulijisikia mwenye wasiwasi?	Sikuwa na wasiwasi kabisa	1
		Nilikuwa na wasiwasi kidogo	2
		Nilikuwa na wasiwasi kiasi (kwa wastani)	3
		Nilikuwa na wasiwasi sana	4
e.	Je, jana ulijisikia mwenye huzuni ?	Sikuwa na huzuni kabisa	1
		Nilikuwa na huzuni kidogo	2
		Nilikuwa na huzuni kiasi (kwa wastani)	3
		Nilikuwa na huzuni sana	4

Ningependa sasa nikuulize maswali kadhaa kuhusu afya yako ya uzazi. Baadhi ya maswali yanaweza kukufedhehesha kuyajibu, Tafadhali kumbuka kwamba taarifa hii ni ya siri.

403	Umewahi kuwa mjamzito tangu ulipofanyiwa mahojiano ya awali (au ya mara ya kwanza) mwanzoni mwa utafiti huu? Mahojiano ya awali yalifanyika [SHOW DATE SHE WAS INTERVIEWED]<br>KAMA NDIYO: Mara ngapi?	Ndiyo<br>Hapana<br>Idadi [ ][ ]	1<br>2	406
404	Umeshazaa watoto wangapi tangu ulipofanyiwa mahojiano ya awali (au ya mara ya kwanza) mwanzoni mwa utafiti huu?	Idadi	[ ][ ]<br>If 00 SKIP TO 406	

405	Watoto wangapi wako hai kati ya hao?<br>WEKA 0 KAMA HAWAPO HAI	Idadi	[ ][ ]	
406	Je, kwa sasa unatumia njia za kisasa za uzazi wa mpango kama vile vidonge vya majira, kitanzi au njia nyingine kama hizo?	Ndiyo<br>Hapana<br>Mjamzito	1<br>2<br>3	
407	Ni jumla ya watu wangapi umejamiiana nao ndani ya miezi 12 iliyopita?<br>WEKA 99 KAMA AMEKATAA KUJIBU.KAMA JIBU NI 00 NENDA SWALI LA 410	Toa idadi ya jumla: [ ][ ]		
408	Ndani ya miezi 12 iliyopita, umewahi kupokea pesa au vitu vingine kwa ajili ya kujamiiana?	Ndiyo<br>Hapana<br>Hakuna jibu	1<br>2<br>99	
409	Ndani ya miezi 12 iliyopita, ulipojamiiana, ni mara ngapi ulitumia condom? Unaweza kusema:<br>SOMA MAJIBU	Karibia kila mara<br>Mara nyingi<br>Mara chache<br>Kama sijatumia kabisa	1<br>2<br>3<br>4	
410	Nisingependa kujua majibu yako, lakini ndani ya miezi 12 iliyopita, umewahi kupima virusi vya UKIMWI?	Ndiyo<br>Hapana<br>Hapana jibu	1<br>2<br>99	
Kuna ongezeko la idadi ya wanawake na wanaume wanaokunywa vinywaji vyenye kilevi nchini Tanzania. Kama hautajali, ningependa kukuuliza kuhusu matumizi yako ya vileo na ya mume/mwenzi wako. Tafadhali, jisikie huru kuwa wazi.				
411	Ndani ya miezi 12 iliyopita, umewahi kunywa kinywaji kilicho na kilevi? Kwa mfano bia, mvinyo, pombe za kienyeji, pombe kali za kienyeji (k.m. Gongo) au vinywaji vinginevyo vyenye kilevi?	Ndiyo<br>Hapana	1<br>2	 415
412	Ni mara ngapi huwa unakunywa kinywaji chenye kilevi? Unaweza kusema:<br>MSOMEE MAJIBU:	Mara 1 mpaka 6 kwa mwaka<br>Mara 2 mpaka 4 kwa mwezi<br>Mara 2 mpaka 3 kwa wiki<br>Mara 4 au zaidi kwa wiki	1<br>2<br>3<br>4	
413	Kwa wastani, huwa unatumia vinywaji vingapi vyenye kilevi katika siku ya kawaida unapokunywa?	1 au 2<br>3 au 4<br>5 au 6<br>7, 8 au 9<br>10 au zaidi	1<br>2<br>3<br>4<br>5	

414		Hapana	Mara moja kwa mwaka	Mara moja kwa kila mwezi	Mara moja kwa kila wiki	Kila siku' karibu kila siku
a.	Huwa unatumia vinywaji sita au zaidi katika tukio moja?<br>KAMA NDIYO, SOMA MAJIBU	1	2	3	4	5
b.	Ndani ya miezi 12 iliyopita uligundua kwamba ilikuwa vigumu kwako kuacha kunywa pombe mara ulipoanza kunywa?<br>KAMA NDIYO, SOMA MAJIBU	1	2	3	4	5
c.	Ndani ya miezi 12 iliyopita ulishindwa kufanya kile ambacho kwa kawaida kilitegemewa kutoka kwako kwa sababu ulikunywa pombe?<br>KAMA NDIYO, SOMA MAJIBU	1	2	3	4	5
d.	Ndani ya miezi 12 iliyopita ulihitaji kuzimua (kunywa kinywaji chenye kilevi kwanza asubuhi) ili uweze kuendelea na shughuli zako baada ya kuwa umekunywa pombe nyingi siku iliyopita<br>KAMA NDIYO, SOMA MAJIBU	1	2	3	4	5
e.	Ndani ya miezi 12 iliyopita ulijihisi kuwa na hatia au ulijuta baada ya kunywa pombe.<br>KAMA NDIYO, SOMA MAJIBU	1	2	3	4	5
f.	Ndani ya miezi 12 iliyopita haukuweza kukumbuka kilichotokea usiku wa siku iliyopita kwa sababu ulikuwa umekunywa pombe?<br>KAMA NDIYO, SOMA MAJIBU	1	2	3	4	5

g.	Je, wewe au mtu mwingine amewahi kujeruhiwa kwa sababu ya unywaji wako wa pombe? – iwe ndani ya miezi 12 iliyopita au kabla ya hapo?	Ndiyo, ndani ya Miezi 12 iliyopita<br>Ndiyo, kabla ya miezi 12 iliyopita<br>Hapana	1<br>2<br>3	
h.	Je, jamaa, marafiki, daktari au watoa huduma wengine wa afya wamekuwa na wasiwasi juu ya unywaji wako wa pombe au wamekushauri upunguze?	Ndiyo, ndani ya miezi 12 iliyopita<br>Ndiyo, kabla ya miezi 12 iliyopita<br>Hapana	1<br>2<br>3	
415	Je, mwenzi wako anakunywa pombe?	Ndiyo<br>Hapana<br>Hana mwenzi/mume	1<br>2<br>3	 417<br>Nenda Sehemu ya 5
416	Ndani ya miezi 12 iliyopita, ni mara ngapi umewahi kumuona mwenzi wako akiwa amelewa pombe?	Haijatokea<br>Mara moja<br>Mara chache<br>Mara nyingi	1<br>2<br>3<br>4	
417	Ndani ya miezi 12 iliyopita, ni mara ngapi umewahi kumuona mwenzi wako akipigana na mtu mwingine ambaye hamuishi naye nyumbani?	Haijatokea<br>Mara moja<br>Mara chache<br>Mara nyingi	1<br>2<br>3<br>4	

SEHEMU YA 5: KUHUSU MITIZAMO NA MILA/DESTURI ZA KIJAMII

Katika jamii hii na mahali pengine popote, watu huwa wana fikra mbalimbali kuhusu familia na juu ya tabia zinazokubalika kwa wanaume na wanawake katika kaya. Tungependa kufahamu maoni yako binafsi juu ya mambo yanayokubalika katika jamii.

	MASWALI	CODING CATEGORIES				
500	Nitakutajia kauli kadhaa kuhusu wanaume na wanawake kwa ujumla. Nitakaposoma kauli zifuatazo tafadhali niambie ni kwa kiasi gani wewe binafsi unakubaliana au kutokubaliana na moja ya kauli hizi<br><br>SOMA SENTENSI, KISHA ULIZA KAMA ANAKUBALIANA AU HAKUBALIANI. HALAFU, ULIZA KAMA ANAKUBALIANA TU AU ANAKUBALIANA KABISA NA KAMA HAKUBALIANI TU, AU HAKUBALIANI KABISA	500<br>Kwa maoni yako binafsi…				
		Ninakubaliana kabisa	Ninakubaliana	Sikubaliani	Sikubaliani kabisa	
a.	Wanandoa wanatakiwa kufanya kwa pamoja maamuzi kuhusu mambo yanayoathiri afya na ustawi wa familia.	1	2	3	4	
b.	Ni wajibu wa mwanamke kujamiiana na mume wake hata kama hataki.	1	2	3	4	
c.	Ni **lazima** mwanaume awe ndiye mtu wa msingi katika kuhudumia familia.	1	2	3	4	
d.	Wanawake wanatakiwa kuwa na haki ya kusoma na kufanya kazi nje ya nyumbani sawa na wanaume.	1	2	3	4	
e.	Mwanamke ni lazima atii matakwa ya mume wake hata kama hakubaliani.	1	2	3	4	
f.	Hata mahusiano yaliyo mazuri yanaweza kujumuisha kupigana ili mradi tu wenzi wanapendana.	1	2	3	4	
g.	Inakubalika kabisa kwa wanawake kufanya kazi nje ya nyumba ili kusaidia familia kiuchumi.	1	2	3	4	
h.	Uongozi wa jamii kwa kiasi kikubwa unatakiwa uwe mikononi mwa wanaume.	1	2	3	4	
i.	Watoto na wanaume wangepata manufaa, kama baba zao wangeshiriki zaidi katika malezi ya watoto wao.	1	2	3	4	
j.	Watoto wa kiume katika familia wanatakiwa wapewe motisha zaidi ya kwenda shule kuliko watoto wa kike	1	2	3	4	
k.	Ni hali ya asili na ni haki kwamba wanaume wana mamlaka zaidi ya wanawake katika familia.	1	2	3	4	
l.	Wanawake wangeweza kuchukua majukumu mengi kuliko wanaume, kama wanaume wangehiari kugawana mamlaka/madaraka.	1	2	3	4	

501	Watu wana maoni tofauti tofauti kuhusu kama kuna mazingira ambapo mwanaume anaweza kufanya ukatili dhidi ya mwenzi wake. Nitakaposoma sentensi zifuatazo, tafadhali sema kama wewe binafsi unakubaliana au haukubaliani.<br>DADISI INAPOBIDI.<br><br>SOMA SENTENSI, KISHA ULIZA KAMA ANAKUBALIANA AU HAKUBALIANI. HALAFU, ULIZA KAMA ANAKUBALIANA TU AU ANAKUBALIANA KABISA<br>AU HAKUBALIANI TU AU HAKUBALIANI KABISA	501 … Kwa maoni yako binafsi…			
		Nakubaliana kabisa	Ninakubaliana	Sikubaliani	Sikubali ani kabisa

a.	Mwanaume ana sababu nzuri ya kumpiga mke wake kama hajakamilisha kazi za nyumbani kwa kiasi kinachoridhisha.	1	2	3	4
b.	Mwanaume ana sababu nzuri ya kumpiga mke wake ikiwa hatamuheshimu.	1	2	3	4
c.	Mwanaume ana sababu nzuri ya kumpiga mke wake iwapo atakataa kujamiiana nae.	1	2	3	4
d.	Mwanaume hana sababu yeyote ya kumpiga mke wake katika hali yeyote ile.	1	2	3	4
e.	Mwanaume ana sababu nzuri ya kumpiga mke wake kama atapinga asiwe na marafiki wengine wa kike	1	2	3	4
f.	Mwanaume ana sababu nzuri ya kumpiga mke wake kama atahisi kuwa si mwaminifu katika ndoa.	1	2	3	4
g.	Mwanaume ana sababu nzuri ya kumpiga mke wake kama atathibitisha kuwa si mwaminifu katika ndoa.	1	2	3	4
h.	Mwanamke ni lazima avumilie ukatili ili kuiweka familia yake pamoja.	1	2	3	4
i.	Ukatili kati ya mume na mke ni suala binafsi hivyo watu wengine hawatakiwi kuingilia kati.	1	2	3	4
j.	Watu wanatakiwa kufanya kila linalowezekana kumsaidia mwanamke aliyeondoka katika mahusiano aliyokuwa anafanyiwa ukatili.	1	2	3	4
k.	Mwanamke aliyebakwa hatakiwi kumwambia mtu yeyote kuhusu suala hilo, la sivyo atalaumiwa yeye mwenyewe kwa shambulizi hilo.	1	2	3	4
l.	Watu wana wajibu wa kuingilia kati kama watasikia au kuona mwanamke akipigwa.	1	2	3	4
m.	Mwanamke ambaye atamwacha mume ambaye huwa anamyanyasa, huleta aibu katika familia yake.	1	2	3	4

502	Sasa ningependa kukuuliza kuhusu maoni yako juu ya tabia za aina fulani. Tafadhali niambie kama wewe unachukulia vitendo vifuatavyo kama aina ya ukatili au unyanyasaji.Tafadhali jisikie huru kuongea ukweli. Una weza ku sema ndiyo, wakati mwingine au hapana.	Ndiyo	Wakati mwingine	Hapana
a.	Mzazi akimpiga kofi mtoto kwa sababu ya kutoheshimu wazee.	1	2	3
b.	Mwanaume akimpiga mke wake kwasababu amemkosea, lakini asimwachie michubuko au alama.	1	2	3
c.	Mwanamke akirudia rudia kumdharau na kumnyanyasa mume wake mbele za watu.	1	2	3
d.	Mzazi akimgombeza mtoto ili asipende kukaa mitaani.	1	2	3
e.	Mwanamke akikataa kujamiiana na mume wake kwa muda wa wiki moja.	1	2	3
f.	Mwanaume akimpiga mwanamke wakati wanabishana, lakini baadaye akaomba msamaha.	1	2	3
g.	Msichana akimwamshia mvulana hisia za kufanya mapenzi lakini asimruhusu kufanya mambo mengine zaidi.	1	2	3
h.	Mwanaume akikataa kumpatia mke wake pesa kwa ajili ya kuendesha familia hata kama ana pesa kwa ajili ya mambo mengine.	1	2	3
i.	Mwanaume akitishia kumpiga mwanamke lakini asimpige kweli.	1	2	3
j.	Mwanaume akilazimisha kujamiiana na mwanamke asiyemfahamu.	1	2	3
k.	Mwanaume akihudumia familia yake lakini akaweka kiasi kingine cha pesa kwa ajili yake binafsi.	1	2	3
l.	Mume akimlazimisha mke wake kujamiiana hata kama mke hataki.	1	2	3

SEHEMU YA 6: KUHUSU UHUSIANO WAKO

KAMA SWALI 201 LIMEJIBIWA HANA MWENZI KATIKA KIPINDI CHA MIEZI 12 ILIYOPITA, ANZIA SWALI LA 607

Pale watu wawili wanapooana, kuishi pamoja au wakiwa katika uhusiano, mara nyingi huwa wanashirikiana katika mambo yote mazuri na mabaya. Ningependa sasa nikuulize maswali kadhaa kuhusu uhusiano wako wa sasa na wa zamani na jinsi mume/mwenzi wako anavyokutendea (alivyokuwa anakutendea). Kama atakuja mtu ghafla tukiwa tunaendelea na mazungumzo, nitabadilisha mada ya mazungumzo. Ningependa tena kukuhakikishia kwamba majibu yako yatatunzwa kwa usiri na kwamba hakutakuwa na ulazima wa kujibu swali lolote ambalo hautapenda kulijibu. Je, ninaweza kuendelea?

	MASWALI	CODING CATEGORIES			
600.	Ndani ya miezi 12 iliyopita, je wewe na mwenzi wako mliwahi kujadili kwa pamoja juu ya mada zifuatazo…..		Kama imewahi kutokea, jaza jibu lake upande wenye kivuli		
		Haijawahi kutokea	Mara moja	Mara chache	Mara nyingi
a.	… mambo yaliyokutokea katika siku? Unaweza kusema haijawahi kutokea, mara moja, mara chache au mara nyingi?	1	2	3	4
b.	… mambo yaliyomtokea yeye katika siku? Unaweza kusema haijawahi kutokea, mara moja, mara chache au mara nyingi?	1	2	3	4
c.	… wasiwasi au hisia ulizokua nazo? Unaweza kusema haijawahi kutokea, mara moja, mara chache au mara nyingi?	1	2	3	4
d.	… wasiwasi au hisia zake? Unaweza kusema haijawahi kutokea, mara moja, mara chache au mara nyingi?	1	2	3	4
601.	Ndani ya miezi 12 iliyopita, je, mwenzi wako aliwahi…		Kama jibu ni ndio, mara ngapi?		
a.	… kukuomba ushauri ili kutatua tatizo lililokuwa linamsumbua? Unaweza kusema haijawahi kutokea, mara moja, mara chache au mara nyingi?	1	2	3	4
b.	… kufuata ushauri uliompatia katika kutatua tatizo lililokuwa linamsumbua? Unaweza kusema haijawahi kutokea, mara moja, mara chache au mara nyingi?	1	2	3	4
c.	… kukusaidia kutafuta kazi? Unaweza kusema haijawahi kutokea, mara moja, mara chache au mara nyingi?	1	2	3	4
d.	… kukutia moyo ushiriki katika shughuli iliyokuwa inafanyika nje ya nyumbani kwa ajili ya faida yako? Unaweza kusema haijawahi kutokea, mara moja, mara chache au mara nyingi?	1	2	3	4
e.	… kukufanya ujisikie unathaminiwa? Unaweza kusema haijawahi kutokea, mara moja, mara chache au mara nyingi?	1	2	3	4

602.	Unajiamini kiasi gani kusimamia maoni yako kama ni tofauti na ya mume wako?<br><br>SOMA MAJIBU	Ninajiamini sana	1
		Ninajiamini lakini ningehitaji kutiwa moyo	2
		Sijiamini hata kidogo	3
		Sifahamu	96
603.	Umesimamia maoni yako ndani ya ya miezi 12 iliyopita?	Ndiyo	1
		Hapana	2

604.	Ungejisikia vizuri kiasi gani kupinga juhudi za mume wako kutaka kutawala baadhi ya mambo katika maisha yako, kama vile nani wa kuonana naye au jinsi ya kutumia pesa?<br><br>SOMA MAJIBU	Vizuri sana<br>Vizuri lakini ningehitaji kutiwa moyo<br>Sio vizuri hata kidogo<br>Sijui	1<br>2<br>3<br>96

605.	Haijalishi wanandoa wanaelewana kiasi gani, kuna wakati huwa hawakubaliani. **Katika uhusiano wako na mwenzi wako wa sasa au wa hivi karibuni....**	Ndiyo	Hapana		
a.	... unaweza kusema mliwahi kuzozana ndani ya miezi 12 iliyopita?<br><br>(NENDA SWALI LA 607 KAMA JIBU NI **HAPANA)**	1	2		
	Mlizozana juu ya……<br><br>KAMA IMEWAHI KUTOKEA, IMETOKEA MARA MOJA, MARA CHACHE AU MARA NYNGI?	HaijawaHi kutokea	Mara moja	Mara chache	Mara nyngi
b.	Shutuma kwamba wewe hautimizi wajibu wako kama mke na kama mama.	1	2	3	4
c.	Yeye (mwenzi) kutokuwa na uwezo au nia ya kutimiza mahitaji ya familia.	1	2	3	4
d.	Masuala mengine ya kifedha na mgawanyo wa rasilimali katika familia.	1	2	3	4
e.	Ulevi/unywaji wake wa pombe/kucheza kamari au kutumia dawa za kulevya.	1	2	3	4
f.	Unywaji wako wa pombe.	1	2	3	4
g.	Wasiwasi kuhusu wenzi wa nje au tuhuma za kutokuwa mwaminifu katika ndoa/mahusiano.	1	2	3	4
h.	Wewe kukataa kujamiiana naye	1	2	3	4
i.	Masuala mengine ya kujamiiana (mara ngapi mnafanya ngono, matumizi ya kondom, n.k.).	1	2	3	4
j.	Wewe kutomtii mume au mwenzi wako au kumkosea heshima.	1	2	3	4
k.	Yeye kutokukuheshimu wewe au watoto wako.	1	2	3	4
l.	Yeye kutofurahishwa na kitendo chako cha kuchukua mkopo kutoka katika shirika linalotoa mikopo midogomidogo ya kifedha.	1	2	3	4

606.	Ndani ya miezi 12 iliyopita, ulipozozana na mume au mwenzi wako, kwa ujumla ulifanya nini?<br>Unaweza kusema ulifanya yafuatayo: haijawahi kutokea, mara moja, mara chache au mara nyingi:		Kama imewahi kutokea,jaza jibu lake upande wenye kivuli		
		haijawaHi kutokea	Mara moja	Mara chache	Mara nyingi
a.	Ulimweleza ulivyojisikia kwa utulivu na heshima.	1	2	3	4
b.	Ulijaribu kumuelewa mwenzi wako na kusikiliza kwa makini kile alichokuwa akisema.	1	2	3	4
c.	Ulimleta mtu au ulijaribu kuleta mtu mwingine ili awasaidie kutatua mambo.	1	2	3	4
d.	Ulipohisi mabishano yamekuwa makali sana, uliondoka ili wewe na mwenzi wako mpate muda wa kutulia.	1	2	3	4
e.	Ulianza kulalamika kuhusu vitu ambavyo vilikuwa havihusiani na mabishano yenu ya awali.	1	2	3	4
f.	Ulimtukana au kumwapizia.	1	2	3	4
g.	Ulipaza sauti na kumfokea	1	2	3	4
h.	Ulimsukuma, mtikisa au kumvuta?	1	2	3	4

607-608	Sasa nitakuuliza kuhusu hali fulani ambazo zinawatokea wanawake walio wengi. Mfikirie mwenzi au mpenzi wako (wa sasa au wa hivi karibuni au wa zamani), je, unaweza kusema kwa ujumla kuwa ni kweli kwamba yeye:	607.		608.<br>ULIZA TU KAMA AMESEMA 'NDIYO KATIKA SWALI LA 607<br>Je, hii imetokea ndani ya miezi 12 iliyopita?	
		Ndio	Hapana	Ndio	Hapana
a.	Anajaribu kukuzuia kuonana na rafiki zako	1	2	1	2
b.	Anajaribu kukuzuia kuwasiliana na familia yako ulikozaliwa	1	2	1	2
c.	Anasisitiza kujua mahali ulipo wakati wote	1	2	1	2
d.	Anaona wivu na anapata hasira ukizungumza na mwanaume mwingine	1	2	1	2
e.	Mara nyingi anakutuhumu kwamba wewe si mwaminifu katika ndoa/mahusiano	1	2	1	2
609 - 610	Mfikirie mume/mwenzi wako (wa sasa au wa hivi karibuni au wa zamani),<br>je, unaweza kusema kwa ujumla kuwa ni kweli kwamba yeye:	609.		610.<br>ULIZA KAMA TU AMESEMA 'NDIYO' KATIKA SWALI LA 609:<br>Je, hii imetokea ndani ya miezi 12 iliyopita?	
a.	Anakataa kukupatia pesa za kutosha kwa ajili ya matumizi ya nyumbani, hata pale anapokuwa na pesa ya kufanyia mambo mengine?	1	2	1	2
b.	Anachukua pesa ambazo umetafuta wewe mwenyewe	1	2	1	2
c.	Hufanya maamuzi muhimu ya kifedha bila kushauriana na wewe	1	2	1	2

Maswali yafuatayo yanahusu mambo yanayowatokea wanawake walio wengi, na kwamba inawezekana mwenzi wako wa sasa au mwingine yeyote wa zamani aliwahi kukufanyia wewe.<br><br>Je, Mume /mwenzi wako wa sasa au mwingine yeyote wa zamani aliwahi….	611.<br>(Kama **NDIYO endelea swali la 612.**<br>**Kama HAPANA nenda swali linalofuata chini ya 611, kama majibu ya maswali yote ni HAPANA nenda swali la 616**)<br><br>NDIYO HAPANA	612.<br>Je, hili limetokea ndani ya miezi 12 iliyopita?<br>(Kama **NDIYO uliza swali la 613 tu. Kama HAPANA uliza swali la 614 tu**)<br><br>NDIYO HAPANA	613.<br>Ndani ya miezi 12 iliyopita unaweza kusema hili limetokea mara moja, mara chache au mara nyingi?<br><br>Moja Chache Nyingi	614.<br>Kabla ya miezi 12 iliyopita unaweza kusema hili limetokea mara moja, mara chache au mara nyingi?<br><br>Moja Chache Nyingi
a. Kukutukana au kukufanya ujisikie vibaya?	1 2	1 2	1 2 3	1 2 3
b. Kukudharau au kukufedhehesha mbele ya watu wengine?	1 2	1 2	1 2 3	1 2 3
c. Kukufanyia vitu vya kuogopesha au kutisha kwa makusudi? (kwa mfano jinsi alivyokuangalia, alikuongelesha kwa sauti ya juu, kuvunja vitu)?	1 2	1 2	1 2 3	1 2 3
d. Kukutishia kwa maneno kukuumiza wewe au mtu mwingine unayemjali?	1 2	1 2	1 2 3	1 2 3

615.<br>KAMA MAJIBU YOTE NI HAPANA KATIKA SWALI 611 USIULIZE SWALI HILI LA 615<br><br>Je, mtu aliyekutukana, kukudhalilisha au kukutisha ni mwenzi wako wa sasa au wa zamani?	Wa sasa 1<br>Wa zamani 2<br>Wote 3

Je, mwenzi wako wa sasa au mwingine yeyote aliwahi….	616.<br>(Kama **NDIYO endelea swali la 617.**<br>**Kama HAPANA nenda swali linalofuata chini ya 616, kama majibu ya maswali yote ni HAPANA nenda swali la 621**)<br><br>NDIYO HAPANA	617.<br>Je, hili limetokea ndani ya ya miezi 12 iliyopita?<br>(Kama **NDIYO uliza swali la 618 tu. Kama HAPANA uliza swali la 619 tu**)<br><br>NDIYO HAPANA	618.<br>Ndani ya miezi 12 iliyopita unaweza kusema hili limetokea mara moja, mara chache au mara nyingi?<br><br>Moja Chache Nyingi	619.<br>Kabla ya miezi 12 iliyopita unaweza kusema hili limetokea mara moja, mara chache au mara nyingi?<br><br>Moja Chache Nyingi
a. kukupiga kofi au kukurushia kitu ambacho kingeweza kukuumiza?	1 2	1 2	1 2 3	1 2 3
b. Kukusukuma, kukutikisa au kukuvuta nywele?	1 2	1 2	1 2 3	1 2 3
c. kukupiga ngumi au kukupiga na kitu chochote ambacho kingeweza kukuumiza	1 2	1 2	1 2 3	1 2 3
d. Alikupiga teke, alikuburuza au alikupiga sana?	1 2	1 2	1 2 3	1 2 3
e. Alikukaba au kukuchoma moto kwa makusudi?	1 2	1 2	1 2 3	1 2 3
f. Alitishia kutumia au alitumia bunduki, kisu au silaha nyingine yoyote dhidi yako?	1 2	1 2	1 2 3	1 2 3

620.<br>KAMA MAJIBU YOTE YA SWALI 616 NI HAPANA,USIULIZE SWALI HILI LA 620<br><br>Je, mtu aliyekufanyia mambo haya ni mwenzi wako wa sasa au wa zamani?			Wa sasa 1<br>Wa zamani 2<br>Wote 3											
	621.<br>(Kama **NDIYO endelea swali la 622.**<br><br>**Kama HAPANA nenda swali linalofuata chini ya 621, Kama majibu ya maswali yote ni HAPANA nenda swali la 626**)		622.<br>Je, hili limetokea ndani ya miezi 12 iliyopita? (Kama **NDIYO uliza swali la 623 tu. Kama HAPANA uliza swali la 624 tu**)		623.<br>Ndani ya miezi 12 iliyopita unaweza kusema hili limetokea mara moja, mara chache au mara nyingi?			624.<br>Kabla ya miezi 12 iliyopita unaweza kusema hili limetokea mara moja, mara chache au mara nyingi?						
	NDIYO	HAPANA	NDIYO	HAPANA	Moja	Chache	Nyingi	Moja	Chache	Nyingi				
a) Je, mume au mwenzi wako wa sasa au mwingine yeyote alishawahi kukulazimisha kujamiianakwa kukutishia, kukushikilia au kukuumiza kwa namna yoyote?	1	2	1	2	1	2	3	1	2	3				
b) Umewahi kujamiiana wakati ulikuwa hautaki kwa sababu ulikuwa unaogopa kwamba mwenzi wako angeweza kukuumiza wewe au mtu mwingine unayemjali kama ungekataa?	1	2	1	2	1	2	3	1	2	3				
c) Umewahi kujamiiana wakati ulikuwa hautaki kwa sababu ulikuwa unaogopa kwamba mume au mwenzi wako angeweza kukuacha au angechukua mwanamke mwingine kama ungekataa?	1	2	1	2	1	2	3	1	2	3				
625.<br>KAMA MAJIBU YOTE YA SWALI 621 NI HAPANA,USIULIZE SWALI HILI LA 625<br><br>Je, mtu aliyekufanyia mambo haya ni mwenzi wako wa sasa au wa zamani?			Wa sasa 1<br>Wa zamani 2<br>Wote 3											
626. Ndani ya miezi 12 iliyopita, wakati ukitendewa matukio ya ukatili, umewahi kujibu mashambulizi ili kujitetea mwenyewe?			Ndiyo 1<br>Hapana 2 (SKIP TO 628)<br>Hana mwenzi ndani ya miezi 12 iliyopita 8 (SKIP TO 632)											
627. KAMA NDIYO: SOMA MAJIBU			Mara moja 1<br>Mara chache 2<br>Mara nyingi 3<br>Sifahamu 4<br>Amekataa kujibu 5											

628.	Ndani ya miezi 12 iliyopita umewahi kumpiga au kumnyanyasa kimwili mume au mwenzi wako wakati yeye alikuwa hakupigi au kukunyanyasa kimwili?	Ndiyo 1<br>Hapana 2 (SKIP TO 630)
629.	KAMA NDIYO: Ni mara ngapi?	Mara moja 1<br>Mara chache 2<br>Mara nyingi sana 3<br>Sijui 4<br>Amekataa kujibu 5

	MASWALI	CODING CATEGORIES		NENDA
630	Ukifikiria siku za nyuma ndani ya miezi 12 iliyopita, unaweza kusema kwamba umewahi kuwa ukimuogopa sana mwenzi wako? Unaweza kusema kuwa haijawahi kutokea, au imetokea mara chache, mara nyingi, kila mara?	Haijawahi kutokea<br>Mara chache<br>Mara nyingi sana<br>Mara kwa mara<br>Amekataa kujibu	1<br>2<br>3<br>4<br>99	
631	Ndani ya miezi 12 iliyopita, watoto wanaoishi katika kaya yako walikuona au kusikia ukipigwa na mwenzi wako? Unaweza kusema, haijawahi kutokea, au imewahi kutokea mara chache, mara kwa mara au mara nyingi sana…	Haijawahi kutokea<br>Mara chache<br>Mara nyingi sana<br>Mara kwa mara<br>Amekataa kujibu<br>Hana watoto	1<br>2<br>3<br>4<br>99<br>96	

Kama NDIYO katika swali lolote kati ya swali ya 611, 616 au 621, endelea. KAMA YOTE NI HAPANA, NENDA SWALI LA 646

Kutokana na majibu yako nimeweza kuona kwamba umewahi kuwa na wakati mgumu na mwenzi wako wa sasa au wa zamani. Sasa ningependa kukuuliza zaidi kuhusu mambo uliyoyafanya ili kushughulikia hali hizi ngumu.

632-633	Nani ulishamwambia kuhusu tabia za mwenzi wako?<br><br>SOMA MAJIBU	632<br><br>Ndiyo	 <br>Hapana	633<br>KATIKA KILA KILICHOTAJWA, ULIZA: uliongea nao ndani ya miezi 12 iliyopita?<br>Ndiyo	 <br>Hapana
a.	Rafiki	1	2	1	2
b.	Wazazi	1	2	1	2
c.	Kaka au dada	1	2	1	2
d.	Mjomba au shangazi	1	2	1	2
e.	Familia ya mume/mwenzi	1	2	1	2
f.	Watoto	1	2	1	2
g.	Majirani	1	2	1	2

h.	Polisi	1	2	1	2
i.	Daktari/mtoa huduma za afya	1	2	1	2
j.	Viongozi wa dini	1	2	1	2
k.	Mshauri nasaha	1	2	1	2
l.	Shirika lisilo la kiserikali/shirika la haki za wanawake	1	2	1	2
m.	Kiongozi wa serikali ya mtaa	1	2	1	2
n.	Mwanachama wa kikundi cha mikopo	1	2	1	2
o.	Mfanyakazi wa BRAC	1	2	1	2
p.	MWINGINE (Taja) ________________________	1	2	1	2
q.	Sijamwambia mtu	1	2		

634 -635	Kuna mtu yeyote aliyejaribu kukusaidia?	634		635 KATIKA KILA KILICHOTAJWA, ULIZA: alikusaidia ndani ya miezi 12 iliyopita?	
		Ndiyo	Hapana	Ndiyo	Hapana
a.	Rafiki	1	2	1	2
b.	Wazazi	1	2	1	2
c.	Kaka au dada	1	2	1	2
d.	Mjomba au shangazi	1	2	1	2
e.	Familia ya mume/mwenzi	1	2	1	2
f.	Watoto	1	2	1	2
g.	Jirani	1	2	1	2
h.	Polisi	1	2	1	2
i.	Daktari/mtoa huduma za afya	1	2	1	2
j.	Viongozi wa dini	1	2	1	2
k.	Mshauri nasaha	1	2	1	2
l.	Shirika lisilo la kiserikali/shirika la haki za wanawake	1	2	1	2
m.	Kiongozi wa serikali ya mtaa	1	2	1	2
n.	Mwanachama wa kikundi cha mikopo	1	2	1	2
o.	Mfanyakazi wa BRAC	1	2	1	2
p.	MWINGINE (Taja)	1	2	1	2
q.	Hakuna aliyenisadia	1	2	1	2

636 -637	Umewahi kwenda sehemu yoyote kati ya zifuatazo kutafuta msaada? SOMA KILA MMOJA	636<br>Ndiyo Hapana	637<br>KATIKA KILA KILICHOTAJWA, ULIZA: Ulienda kutafuta msaada sehemu hizi ndani ya miezi 12 iliyopita?<br>Ndiyo Hapana
a.	Polisi	1 2	1 2
b.	Hospitali au kituo cha afya	1 2	1 2
c.	Huduma za jamii	1 2	1 2
d.	Kituo cha huduma za kisheria	1 2	1 2
e.	Mahakamani	1 2	1 2
f.	Makazi ya watu waliopata matatizo	1 2	1 2
g.	Kiongozi wa mtaa	1 2	1 2
h.	Shirika la wanawake (Taja jina)	1 2	1 2
i.	Mchungaji/kiongozi wa dini	1 2	1 2
j.	Sehemu nyingine yoyote? Wapi/Taja?_____ _____________	1 2	1 2

638	Ulishawahi kuondoka, hata kama ni kwa usiku mmoja tu, kwa sababu ya tabia yake?	Ndiyo<br>Hapana	1<br>2	 645
639	Kama ndiyo, mara ngapi?	Idadi [ ] [ ]		
640	Je, uliwahi kuondoka hata angalau usiku mmoja ndani ya miezi 12 iliyopita	Ndiyo<br>Hapana	1<br>2	 645
641	Je, ulikaa mbali kwa muda gani **mara ya mwisho ulipoondoka?**	Siku moja<br>Siku kadhaa<br>Miezi<br>Niliachana na mwenzi	1<br>2<br>3<br>4	
642	Ulikwenda wapi?	Kwenye familia<br>Rafiki/Jirani<br>Hotelini<br>Nyingine Taja	1<br>2<br>3<br>4	
643	Umesha rudi?	Ndio<br>Hapana	1<br>2	 645
644	[KAMA ALIRUDINYUMBANI] Kwa nini ulirudi?	Sikutaka kuwaacha watoto<br>Utakatifu wa ndoa<br>Niliambiwa na familia yangu<br>Sikufahamu jinsi gani nitajihudumia mwenyewe<br>Nilimsamehe<br>Alinitishia<br>Nyingine Taja_...................________________	1<br>2<br>3<br>4<br>5<br>6<br>7	

645	Ndani ya miezi 12 iliyopita, kwa ujumla, ni kwa namna gani kuwa mwanachama wa BRAC kumekuwezesha kukabiliana na hali ya unyanyasaji wa mwenzi wako? Unaweza kusema kwamba:	Haijaathiri kitu<br>Imefanya hali iwe mbaya<br>Imeboresha hali<br>Hana mwenzi<br>Amekataa kujibu<br>Sio mwanakikundi wa BRAC	1<br>2<br>3<br>4<br>96<br>99	

Wanawake wengi huwa hawajisikii vizuri kujadili uzoefu kama huu na wakati mwingine hawapendi kuweka wazi kila kitu kuhusu mahusiano yao na uzoefu wao. Inawezekana kuna sababu nyingine ambazo zinamfanya mwanamke asiweke wazi taarifa ambazo nimekwisha kuuliza na hiyo ni sawa. Unaweza kukataa kujibu baadhi ya maswali kama hutajisikia vizuri. Ningependa kuuliza baadhi ya maswali ili nielewe jinsi unavyojisikia ukilinganisha na wakati ulipofanyiwa mahojiano ya awali (au ya mara ya kwanza) mwanzoni mwa utafiti huu. Mahojiano ya awali yalifanyika [SHOW DATE SHE WAS INTERVIEWED]				
646	Ukilinganisha na mahojiano ya awali (au ya mara ya kwanza) tuliyokufanyia mwanzoni mwa utafiti huu, unaweza kusema unajisikia vizuri zaidi au hujisikii vizuri kujadili uhusiano wako binafsi haswa uzoefu ambao tumemaliza kuzungumzia hivi punde?	Najisikia vizuri zaidi<br>Hakuna mabadiliko (ni kama sawa sawa na mahojianao ya mara ya mwisho)<br>sijisikii vizuri zaidi	1<br>2<br>3	
647	Wakati wa mahojiano ya awali (au ya mara ya kwanza) mwanzoni mwa utafiti huu, unaweza kusema kwamba ulitoa taarifa za uzoefu wako kwa usahihi?	Ndiyo – nilieleza kila kitu<br>Hapana – sikueleza kila kitu<br>Sina hakika/sikumbuki	1<br>2<br>3	
648	Wakati wa mahojiano ya awali (au ya mara ya kwanza) mwanzoni mwa utafiti huu, kulikuwa na baadhi ya uzoefu ambao haukupenda kuujadili au kuuweka wazi?	Ndiyo - sikupenda kujadili/kuweka wazi baadhi ya uzoefu<br>Hapana – nilijisikia kujadili/kuweka wazi uzoefu wote<br>Sikumbuki	1<br>2<br>3	
649	Ukifikiria kuhusu uzoefu kuhusu mizozo/magomvi katika mahusiano ambao tumemaliza hivi punde kuujadili, ukilinganisha na wakati wa mahojiano ya awali (au ya mara ya kwanza) mwanzoni mwa utafiti huu, unaweza kusema kwamba huu uzoefu ni katika kiwango kama kile kile, umepungua na kuwa ni wa mara chache au umeongezeka na kuwa ni mara kwa mara?	Umepungua nikilinganisha na awali<br>Karibu uko sawa sawa (hakuna mabadiliko)<br>Umeongezeka na kuwa mara kwa mara	1<br>2 (SKIP TO 651)<br>3	
650	kama mshiriki ameashiria/ameonyesha mabadiliko katika uzoefu wake (kuongezeka au kupungua) - muulize... Unafikiri mabadiliko hayo ni kwa sababu umeshiriki katika utafiti wa MAISHA?	Ndiyo<br>Hapana<br>Sijui	1<br>2<br>3	

		Mshiriki		Mwenzi wako au mtu mzima mwingine katika kaya	
651	**Kama swali 631 limejibiwa hana mtoto, nenda sehemu ya 7** Watu wazima hutumia njia fulani kuwafundisha watoto tabia njema au kutatua tatizo la tabia. Nitakusomea njia mbalimbali ambazo hutumika. Tafadhali niambie kama wewe,mwenzi wako au mtu mzima mwingine yeyote katika kaya yako ametumia njia hii kwa mtoto yeyote katika kaya yako ndani ya mwezi mmoja uliopita. Onyesha njia moja au zaidi ambayo wewe, mwenzi au mtu mzima mwingine ameitumia	Ndiyo	Hapana	Ndiyo	Hapana
a.	Kuwaondolea vitu wanavyovipendelea, kuwakataza kitu walichokipenda au kumzuia mtoto asitoke nyumbani?	1	2	1	2
b.	Kumuelezea mtoto kwa nini tabia yake siyo sahihi	1	2	1	2
c.	Kumtikisa mtoto kwa nguvu	1	2	1	2
d.	Kupaza sauti, au kupiga kelele na kumfokea?	1	2	1	2
e.	Kumpa kitu kingine cha kufanya?	1	2	1	2
f.	Kumchapa matakoni kwa kutumia mkono bila kitu kingine	1	2	1	2
g.	Kumpiga matakoni au sehemu nyingine ya mwili kwa kutumia kitu kama mkanda, brashi ya viatu au kitu kigumu	1	2	1	2
h.	Kumuita kuwa hana akili, mvivu au majina mengine kama hayo	1	2	1	2
i.	Kumpiga usoni, kichwani au masikioni	1	2	1	2
j.	Kumpiga kofi kwenye kiganja cha mkono, mkononi, au mguuni	1	2	1	2
k.	Kumpiga sana yaani kumpiga tena na tena kwa nguvu kadri alivyoweza	1	2	1	2
l.	Je ,Unaamini kwamba, ili kumkuza/kumlea mtoto vizuri au kumuelimisha kwa usahihi, inabidi mtoto apigwe (au aadhibiwe kwa kupigwa mwilini)?	1	2	1	2

SEHEMU YA 7: KUHUSU KIPINDI CHA UTOTO WA MWENZI WAKO

TAFADHALI ANGALIA SWALI LA 201, KAMA JIBU NI HAPANA – ULIZA SWALI LA 700 – 704 KUHUSU MWENZI WAKE WA SASA ALIPOKUWA MDOGO, KAMA JIBU NI NDIO, NENDA SEHEMU YA 8
Ningependa nikuulize maswali machache ambayo inawezekana yalitokea kwa mwenzi wako wa sasa alipokuwa mtoto

700	Kwa kadri unavyojua, wakati mwenzi wako wa sasa alipokuwa mtoto, aliwahi kuishi na mlevi katika kaya,?	Ndiyo<br>Hapana<br>Sijui<br>Sina mwenzi	1<br>2<br>96<br>99
701	Kwa kadri unavyojua, wakati mwenzi wako wa sasa alipokuwa mtoto, aliwahi kuishi na mtu aliyewahi kufungwa jela?	Ndiyo<br>Hapana<br>Sijui<br>Sina mwenzi	1<br>2<br>96<br>99
702	Kwa kadri unavyojua, wakati mwenzi wako wa sasa alipokuwa mtoto, mama au baba yake au mlezi wake alifariki?	Ndiyo<br>Hapana<br>Sijui<br>Sina mwenzi	1<br>2<br>96<br>99
703	Kwa kadri unavyojua, wakati mwenzi wako wa sasa alipokuwa mtoto, alikuwa anapigwa mara kwa mara na mtu katika familia yake?	Ndiyo<br>Hapana<br>Sijui<br>Sina mwenzi	1<br>2<br>96<br>99
704	Kwa kadri unavyojua, wakati mwenzi wako wa sasa alipokuwa mtoto, alikuwa anapigwa sana mpaka kuachiwa alama mwilini au kujeruhiwa?	Ndiyo<br>Hapana<br>Sijui<br>Sina mwenzi	1<br>2<br>96<br>99

SEHEMU YA 8: KUHUSU JAMII YAKO

Ningependa kufahamu zaidi kuhusu vikundi au mashirika ambayo wewe ni mshiriki kutoka katika jamii yako.

	MASWALI	CODING CATEGORIES				
800-801	Tafadhali niambie kama wewe ni mshiriki wa kikundi kimojawapo au vikundi zaidi ya kimoja kati ya vikundi vifuatavyo.	800.		801.<br>KAMA **NDIYO KWA SWALI 800**, tafadhali niambie pia kama huwa unahudhuria tu, kama ni mwanachama hai au kama ni kiongozi wa mojawapo ya shughuli za vikundi hivi.		
		Ndiyo	Hapana	Mwanachama/ anahudhuria tu	Hai	Kiongozi
a.	Vikundi vya kidini	1	2	1	2	3
b.	Vikundi vya kikabila/kijadi	1	2	1	2	3
c.	Vikundi vya kusaidiana kiuchumi (visivyo vya -kifedha)	1	2	1	2	3
d.	Vikundi vya mtaa	1	2	1	2	3
e.	Vikundi vya sherehe/ kuzikana	1	2	1	2	3
f.	Vikundi vya vijana	1	2	1	2	3
g.	Vikundi vya wanawake (visivyo vya -kifedha)	1	2	1	2	3
h.	Kamati ya shule	1	2	1	2	3
i.	Kamati ya afya	1	2	1	2	3
j.	Vikundi vya michezo	1	2	1	2	3
k.	Vikundi vya mikopo	1	2	1	2	3
l.	Wanasheria/ Vikundi vya kitaaluma	1	2	1	2	3
m.	Vikundi vya wanaharakati	1	2	1	2	3
n.	Vingine (Taja)					

	MASWALI	CODING CATEGORIES	
802.	Sasa nitakuuliza maswali kadhaa kuhusu namna jamii inavyofanya kazi na jinsi inavyoshughulikia matatizo.<br>Ikitokea watu wawili katika kijiji au mtaa huu wakawa na mgogoro mkubwa baina yao. Unafikiri ni nani atasaidia kutatua mgogoro huo?	Hakuna mtu/wahusika watasuluhisha wao kwa wao	1
		Familia/wanakaya	2
		Majirani	3
		Wanakikundi	4
		Viongozi wa jamii	5
		Viongozi wa dini	6
		Viongozi wa kisheria	7
		Nyingine,Taja.	8

803.	Ndani ya miaka 2 iliyopita, umewahi kushiriki katika mikutano, maandamano au mkusanyiko uliolenga kuongeza uelewa na kuwahamasisha watu juu ya suala muhimu katika jamii yako? kwa mfano Virusi Vya UKIMWI, haki za watu wenye ulemavu wa ngozi au wanawake n.k	Ndiyo<br>Hapana	1<br>2 (SKIP TO 806)
804.	Umewahi kushiriki katika kuandaa mkutano au mkusanyiko kama huo?	Ndiyo<br>Hapana	1<br>2 (SKIP TO 806)
805.	Kama **NDIYO,** aina gani ya mkutano?	Taja:___________________	
806.	Mara nyingi watu wanaona aibu kuzungumza mbele za watu. Kama ungekuwa katika mkutano wa jamii (kwa mfano, kamati ya shule), unajiamini kiasi gani kwamba wewe unaweza kutoa maoni yako mbele za watu? Je, unaweza kusema .... (SOMA MAJIBU)	Unajiamini sana<br>Unajiamini, lakini ungehitaji kutiwa moyo<br>Hujiamini hata kidogo<br>Hujui	1<br>2<br>3<br>99
807.	Umewahi kuongea mbele za watu ndani ya miezi 12 iliyopita?	Ndiyo<br>Hapana	1<br>2
808.	Mara nyingi majirani wana matatizo yanayofanana (kwa mfano, kuhusu malezi ya watoto). Unajiamini kiasi gani kwamba unaweza kutoa ushauri kwa jirani au rafiki yako? Je, unaweza kusema: (SOMA MAJIBU)	Unajiamini sana<br>Unajiamini, lakini unahitaji kutiwa moyo<br>Hujiamini hata kidogo<br>Hujui	1<br>2<br>4<br>99
809.	Umetoa ushauri kama huo ndani ya miezi 12 iliyopita?	Ndiyo<br>Hapana	1<br>2
810.	Kama ungefanyiwa vitendo vibaya na mume au mpenzi wako, ungejisikia vizuri kiasi gani kutafuta msaada kutoka kwa rafiki unayemuamini au jirani? Je, unaweza kusema:	Vizuri sana<br>Vizuri lakini ningehitaji kutiwa moyo<br>Sio vizuri hata kidogo<br>Sijui	1<br>2<br>3<br>99
811.	Unajiamini kiasi gani juu ya uwezo wako wa kuongea katika kumtetea mwanamke aliyefanyiwa ukatili wa kingono? Je, unaweza kusema:	Unajiamini sana<br>Unajiamini, lakini unahitaji kutiwa moyo<br>Hujiamini hata kidogo<br>hujui	1<br>2<br>3<br>99
812.	Unajiamini kiasi gani katika uwezo wako wa kuingilia kati kesi za ukatili majumbani?	Unajiamini sana<br>Unajiamini, lakini unahitaji kutiwa moyo<br>Hujiamini hata kidogo<br>Hujui	1<br>2<br>3<br>99
813.	Je, uliingilia kati kesi za ukatili majumbani ndani ya miezi 12 iliyopita?	Ndiyo<br>Hapana	1<br>2

SEHEMU YA 9:

Kabla ya kumaliza mahojiano yetu, ningependa nikuulize maswali machache kuhusu mafunzo ya jinsia katika utafiti huu wa MAISHA

	MASWALI	CODING CATEGORIES	NENDA
900.	Je, uliwahi kuhudhuria kipindi chochote cha mafunzo ya jinsia ambayo yalitolewa katika utafiti huu wa MAISHA	Ndiyo 1<br>Hapana 2	 903
901.	Katika utafiti wa MAISHA mafunzo ya jinsia yalitolewa katika vipindi kumi. Je, wewe ulihudhuria vipindi vingapi?<br><br>Naomba ufikirie kwa makini ili unipe jibu sahihi	IDADI YA VIPINDI [ ] [ ]<br>Kama hakumbuki andika 99	
902.	Je, ni mafunzo gani uliona yamekusaidia zaidi wewe binafsi na kwenye mahusiano yako?<br>(andika mafunzo yote atakayokumbuka kuwa yalimsaidia)<br><br>Je, kuna kitu kingine mafunzo hayo yalikusaidia?<br>1 Ndiyo 2 Hapana<br><br>Kama Hapana end of probe		
903	Tangu ulipojiunga katika utafiti wa MAISHA, umewahi kujadili masuala kama vile ukatili dhidi ya wanawake au jukumu la wanawake katika jamii na wanawake wanaoshiriki katika utafiti?	Haijawahi kutokea 1<br>Mara chache 2<br>Mara nyingi sana 3<br>Mara kwa mara 4<br>Amekataa kujibu 5	
904	Tangu ulipojiunga katika utafiti wa MAISHA, umewahi kujadili masuala kama vile ukatili dhidi ya wanawake au jukumu la wanawake katika jamii na wanawake ambao hawakushiriki katika utafiti?	Haijawahi kutokea 1<br>Mara chache 2<br>Mara nyingi sana 3<br>Mara kwa mara 4<br>Amekataa kujibu 5	
905	Je, utapenda tuwasiliane nawe siku zijazo kwa ajili ya mambo mbali mbali yanayohusu utafiti huu wa MAISHA	Ndiyo 1<br>Hapana 2	

MASWALI	CODING CATEGORIES	NENDA

MAISHA: Utafiti wa uhusiano
na Afya
Dodoso la wanawake

UTAKAPOMALIZA

Maelezo ya mahojiano – MWISHO

Tarehe ya mahojiano: **Siku[ ] [ ] /Mwezi[ ] [ ] [ ]/ Mwaka [ ] [ ] [ ] [ ]**

Muda wa kumaliza mahojiano: **[ ] [ ]: [ ] [ ]**

Jina la mhojaji: **[ ] [ ] [ ]**

Je, wewe ni mhojaji yule aliyeanza mahojiano toka mwanzo? 1. Ndiyo 2 Hapana

Maoni:

Survey of men

Lengo la utafiti wa maisha ni kutathimini njia mbalimbali za kuboresha mahusiano, na afya kwa ujumla. Kama unavyofahamu tulikuwa tunafanya kazi na makundi ya wanawake wanaochukua mikopo midogo midogo, sasa tungependa kufahamu mitazamo ya wanaume kuhusu mahusiano mazuri. Tumepanga kufanya mahojiano mafupi na wenzi wa wanawake ambao wamo katika utafiti wa MAISHA. Wakati wa mahojiano hayo, tutamuuliza mwanaume maswali kuhusu yeye mwenyewe, afya yake na uzoefu binafsi katika mahusiano. Tungependa kufahamu kama unaridhia mtafiti wa jinsia ya kiume wa timu MAISHA kuwasiliana na mwenzi wako na kumkaribisha kushiriki mahojiano hayo. Hututawasiliana naye kama haujaridhia. Kama utaridhia sisi kuwasiliana na mwenzi wako sasa lakini baadaye ukabidilisha mawazo hilo pia sio tatizo- wasiliana nasi kwa namba <INGIZA NAMBA> kutufahamisha.

Iwapo mwenzi wako ataridhia kushiriki katika utafiti, ataombwa kuhudhuria mahojianokwa mara moja tu, ambayo yatafanyika mwezi mmoja kutoka sasa, kwa wakati na mahali ambapo atapendelea. Mahojiano yatafanywa na mtafiti wa jinsia ya kiume na yatatumia takribani saa moja.

Iwapo utapenda, unaweza kuongea na mwenzi wako kwanza kuhusu utafiti huu ili kujua kama angependelea kushiriki na kama atakubali kwa mtafiti kuwasiliana naye. Vinginevyo, kama utapenda kunipatia namba ya mkononi ya mwenzi wako na taarifa zake za mawasiliano, mtafiti mmojawapo wa jinsia ya kiume atafanya mawasiliano na mwenzi wako moja kwa moja mwezi mmoja kutoka sasa, ili kupanga muda wa kukutana na kujadili kuhusu utafiti huu. Tutakufahamisha kabla ya kuwasiliana naye iwapo utakua umebadili mawazo.

Unadhani mwenzi wako atapendelea kushiriki? 1. Ndiyo 2. Hapana 3. Sijui 4. Hakuna jibu
Iwapo jibu ni ndio,

Ungependelea kuzungumza na mwenzi wako kwanza? 1. Ndiyo 2. Hapana 3. Sijui 4. Hakuna jibu

Unaridhia kwa mtafiti wa MAISHA kuwasiliana naye mwezi mmoja kutoka sasa? 1. Ndiyo 2. Hapana 3. Sijui 4 Hakuna jibu

Iwapo jibu ni ndio, tafadhali kamilisha maelezo hapa chini:

Jina la mwenzi:

Namba ya simu ya mkononi ya mwenzi:

Taarifa zingine za mawasiliano za mwenzi (mf. kazini):

Kufunga mahojiano

Namna ya kwanza – Kama mshiriki ameeleza wazi kwamba amekumbana na matatizo/ukatili

.

Ningependa nikushukuru sana kwa kutusaidia. Ninashukuru sana kwa muda ulioutumia. Nimegundua kuwa inawezekana haya maswali yalikuwa magumu kwako kuyajibu, lakini ni kwa njia ya kuongea na wanawake wenyewe tu ndipo tunaweza tu kufahamu ukweli juu ya hali walizonazo na jinsi ya kuwasaidia.

Kutokana na yale uliyotueleza, ninaweza kufahamu kwamba umekumbana na matatizo mengi katika maisha yako. Hakuna mtu mwenye haki ya kumtisha mtu mwingine kiasi hicho. Hata hivyo, kutokana na vile ulivyonielezea nimeweza kufahamu kwamba wewe ni mtu jasiri sana na umeweza kupitia mambo magumu.
Hapa tuna orodha ya mashirika ambayo yanatoa msaada, ushauri wa kisheria na huduma ya ushauri kwa wanawake hapa Mwanza. Tafadhali wasiliana nao kama ungependa uzungumze na mtu yeyote kuhusu matatizo yako. Huduma zao zinatolewa bure, na watatunza mambo yote utakayowaambia kwa usiri mkubwa. Unaweza kwenda wakati wowote utakapokuwa tayari hivi karibuni au baadaye. Tutafurahi kukusaidia kuwasiliana nao kama unaona hii itakusaidia.

Namna ya pili – Mshiriki hajaeleza wazi matatizo/ukatili

Ningependa nikushukuru sana kwa kutusaidia. Ninashukuru sana kwa muda ulioutumia. Nimegundua kuwa inawezekana haya maswali yalikuwa magumu kwako kuyajibu, lakini ni kwa njia ya kuongea na wanawake wenyewe tu ndipo tunaweza tu kufahamu ukweli juu ya hali zao za kiafya na matukio mbalimbali wanayokumbana nayo wanawake katika maisha.

Iwapo utasikia mwanamke mwingine ambaye anahitaji msaada, hapa tuna orodha ya mashirika ambayo yanatoa msaada, ushauri wa kisheria na huduma ya ushauri kwa wanawake hapa Mwanza. Tafadhali wasiliana nao kama wewe au rafiki zao au ndugu yako atahitaji msaada. Huduma zao zinatolewa bure, na watatunza mambo yote utakayowaambia kwa usiri mkubwa.
