# Supplementary material for: Couples data from north-western Tanzania: Insights from a survey of male partners of women enrolled in the MAISHA cluster randomized trial of an intimate partner violence prevention intervention
Source: PLoS One. 2020 Oct 2;15(10):e0240112. doi: 10.1371/journal.pone.0240112 (PMC7531846; doi:10.1371/journal.pone.0240112)
Supplement: S3 Questionnaire — (DOCX) [file pone.0240112.s003.docx]

MALE PARTNER QUESTIONNAIRE

CONFIDENTIAL UPON COMPLETION

SECTION 1: ABOUT YOU 4

SECTION 2: ABOUT YOU AND YOUR HEALTH 7

SECTION 3: ABOUT ATTITUDES AND SOCIAL NORMS 9

SECTION 4: ABOUT YOUR RELATIONSHIP 10

SECTION 5: ABOUT YOUR CHILDREN 12

SECTION 6: ABOUT CHILDHOOD 13

SECTION 7: ABOUT YOUR COMMUNITY 15

| **Introduction**  Hello, my name is _____________________ , I am from the National Institute for Medical Research/Mwanza Interventions Trial Unit. As you know, you have agreed to take part in the study we are currently conducting in Mwanza. As part of this study we would like to ask you a number of questions about yourself, your household, your relationship and your community. Some of the questions are sensitive, but everything that you say will be kept private, and will not be shared with others. I would like to encourage you to be honest, as there are no right or wrong answers. At any point you can stop the interview, or choose to not answer a question. This will not affect your further involvement in the study. If you find anything that we discuss upsetting, and would like to talk to someone afterwards, we can help with this.  The interview will take two hours or more to complete. For this it is best if we are not interrupted. Is this a good place to talk, or should we go somewhere else where we can talk privately?  Do you have any questions? |
| --- |

**BEFORE YOU START**

| **Participant Identification Number** | | |
| --- | --- | --- |
| **Female** ID | **[ ] [ ] [ ] [ ] - [ ] [ ] [ ] [ ] - [ ] [ ]** | |
| **PLEASE USE THE FEMALE ID WITH THE IDENITIFCAITON LIST TO IDENTIFY THE WOMAN. THEN CONFIRM THAT THE MAN YOU ARE INTERVIEWING IS IN A RELATIONSHIP WITH THIS WOMAN.** | | |
| **Yes**  In relationship with this (female ID) woman.  Continue with the questionnaire | | **No**  Not in a relationship with this (Female ID) woman.  Stop the interview and inform the team leader. |

**! PLEASE FILL IN COMPLETE PARTICIPANT ID AT THE BOTTOM OF EVERY PAGE OF THIS QUESTIONNAIRE !**

| **Interview details – START**  Date of interview: **[ ] [ ] /[ ] [ ] [ ]/ [ ] [ ] [ ] [ ] dd/MMM/yyyy**  Time interview started: **[ ] [ ]: [ ] [ ]**  Name of interviewer: **[ ] [ ] [ ]** |
| --- |

| SECTION 1: ABOUT YOU |
| --- |
|  |

I would now like to ask you a few questions about yourself, the kind of work you are doing and how you feel or have felt about some things that have happened to you.

|  | QUESTIONS | CODING CATEGORIES | |
| --- | --- | --- | --- |
| 100 | How old are you?  GET RESPONDENT TO ESTIMATE IF DON'T KNOW EXACTLY | Years: [ ][ ] | |
| 101 | What is your date of birth?  (RECORD AS MUCH INFORMATION AS POSSIBLE. CODE 96 IF DAY UNKNOWN, 969 IF MONTH UNKNOWN, AND 9696 IF YEAR UNKNOWN) | DAY [ ][ ]  MONTH ( ) ( )( )  YEAR [ ][ ] [ ][ ] | |
| 102 | How long have you been living in Mwanza? | Years: [ ][ ] Months: [ ][ ] |  |
| 103 | Are you of Tanzanian nationality? | Tanzanian  Not Tanzanian | 1  2 |
| 104 | Where did you grow up?  PROBE IF NEEDED: Before age 12, where did you live the longest? | This community/neighbourhood  Another rural area/village in Tanzania  Another town/city in Tanzania  Another country  Don’t know/don’t remember  Refused/no answer | 1  2  3  4  96  99 |
| 105 | Who did you grow up with? | Both mother and father  Mainly mother  Mainly father  Other. Please specify:_______________ | 1  2  3  4 |
| 106 | How would you describe your religious orientation? | Muslim  Seventh day Adventist  Tanzanian Assemblies of God  Catholic  Lutheran  Moravian  Pentecostal  African Inland Church  No religion  Other (Specify): . . . . . . . . . . . . . . . . . . . | 1  2  3  4  5  6  7  8  9  10 |
| 107 | What is your ethnic background? | Msukuma  Mjita  Mzinza  Myiramba  Mkara/Mkerewe  Mhaya  Mjaluo  Mkuria/Mshashi  Mchaga  Mhindi  Mwarabu  Other (Specify):. . . . . . . . . . . . . . . . . . . | 1  2  3  4  5  6  7  8  9  10  11  12 |
| 108 | What is the highest level of schooling you ever completed? | Never went to school  Primary incomplete  Primary complete  Secondary incomplete  Secondary (Form I-IV)  Secondary (Form V-VI)  College training after primary/secondary school and before university  University | 1  2  3  4  5  6  7  8 |

I would now like to find out a bit more about the ways that you earn money.

| 109 | What is the main sources of income for you and your family?  CAN ALLOW MULTIPLE RESPONSES | Yes | No |  |
| --- | --- | --- | --- | --- |
| A | Money from own work | 1 | 2 |  |
| B | Support from wife/partner | 1 | 2 |  |
| C | Support from other relatives | 1 | 2 |  |
| D | Pension | 1 | 2 |  |
| E | Social services/welfare | 1 | 2 |  |
| F | Other - specify | 1 | 2 |  |
| 110 | Have you personally earned money during the past 12 months? | Yes  No | 1  2 | 220 |
| 111 | Are you self-employed or do you work for someone else/organisation? | Self employed  Work for someone else or an organization  Both are true | 1  2  3 |  |
| 112 | Is this a regular or an occasional activity? | Regular  Occasional | 1  2 |  |
| 113 | On a typical working day, how many hours do you work?  ENTER 96 IF DON’T KNOW | Hours: [ ] [ ] | |  |
| 114 | On a typical working day/week/month how much do you earn from this activity?  FILL OUT EITHER DAY, WEEK OR MONTH | Day: [ ] [ ] [ ] [ ] [ ] [ ]  Week: [ ] [ ] [ ] [ ] [ ] [ ]  Month: [ ] [ ] [ ] [ ] [ ] [ ] | |  |

|  |  |  | If **notNEVER**… | | |
| --- | --- | --- | --- | --- | --- |
| 115 | Nowadays, many families have a hard time making ends meets. I would like to learn more about how your household is coping.  EMPHASIZE THAT YOU ARE TALKING ABOUT THE PAST **12 MONTHS**  **In the past 12 months…….** | Never | Once | Few times | Many times |
| A | … were you very worried/stressed about your general financial situation.  Would you say ,this has happened or never happened? | 1 | 2 | 3 | 4 |
| B | … have you had trouble buying food or other necessities for your family?  Would you say ,thishas happened or never happened? | 1 | 2 | 3 | 4 |
| C | … have you had to borrow money to pay rent or other bills ?  Would you say ,this has happened or never happened? | 1 | 2 | 3 | 4 |
| D | … did any of your family members need to see a doctor but could not because you did not have enough money?  Would you say ,this has happened or never happened? | 1 | 2 | 3 | 4 |
| E | … did your children miss days of school because you did not have money for school fees, uniforms or supplies?  Would you say ,this has happened or never happened? | 1 | 2 | 3 | 4 |
| F | … have you or any of your own children gone a whole day without eating anything because there was not enough food?  Would you say ,this has happened or never happened? | 1 | 2 | 3 | 4 |

**Partner’s micro-finance participation**

| 116 | In the past 12 months,has your partner ever borrowed money from a microfinance provider, different from BRAC, like SACCOS, FINCA, PRIDE or others? | Yes  No | 1  2 | 200 |
| --- | --- | --- | --- | --- |
| 117 | How confident do you feel about your partner being able to pay this back? Would you say that you feel:  Very confident, Somewhat confident, Not very confident? | Very confident  Somewhat confident  Not very confident | 1  2  3 |  |
| 118 | How important is the money that your partner gets from micro-finance for the family?  Is it extremely important, somewhat important, or not very important at all? | Very important  Somewhat important  Not very important | 1  2  3 |  |
| 119 | In the past 12 months, overall, how has your partner being a member of a microfinance group affected your relationship with your partner? Would you say that itshas:= had not effect on the relationship, made your relationship more difficult, or improved your relationship? | Not affected the relationship  Made your relationship more difficult  Improved your relationship | 1  2  3 |  |

| SECTION 2: ABOUT YOU AND YOUR HEALTH |
| --- |
|  |

I would now like to ask you several questions about your health.

|  |  |  | |
| --- | --- | --- | --- |
| 200 | During the last 4 weeks, have you been bothered by any of the following problems? | Yes | No |
| a | Do you often have headaches? | 1 | 2 |
| b | Is your appetite poor? | 1 | 2 |
| c | Do you sleep badly? Like difficulties falling asleep, waking up in the middle of the night more than 3 times or waking up early in the morning and not getting back to sleep. | 1 | 2 |
| d | Are you easily frightened? | 1 | 2 |
| e | Do your hands shake? | 1 | 2 |
| f | Do you feel nervous, tense or worried? Like thinking of something in more detail | 1 | 2 |
| g | Is your digestion poor? Like you are often constipated, feel nauseous, or you don’t have an appetite. | 1 | 2 |
| h | Do you have trouble thinking clearly? | 1 | 2 |
| i | Do you feel unhappy? | 1 | 2 |
| j | Do you cry more than usual? Like every day or under normal circumstances most people will not cry, because of problems or various incidents in life? | 1 | 2 |
| k | Do you find it difficult to enjoy your daily activities? | 1 | 2 |
| l | Do you find it difficult to make any decisions? | 1 | 2 |
| m | Is your daily work suffering? | 1 | 2 |
| n | Are you unable to play a useful part in life? | 1 | 2 |
| o | Have you lost interest in things? | 1 | 2 |
| p | Do you feel that you are a worthless person? | 1 | 2 |
| q | Has the thought of ending your life been on your mind? | 1 | 2 |
| r | Do you have uncomfortable feelings in your stomach? | 1 | 2 |
| s | Are you easily tired? | 1 | 2 |

I would now like to ask you several questions about your sexual health. Some of the questions may be embarrassing to answer. Please remember that this information is confidential and you may choose not to answer them.

| 201 | At what age did you first have sexual intercourse? | | Age in years: [ ][ ]  96 if never had sex  99 If don’t remember | | | |  | | 206  206 |
| --- | --- | --- | --- | --- | --- | --- | --- | --- | --- |
| 202 | How many people in total have you had sexual intercourse with in your life? | | Give total number:  99 If don’t rememember | | | | [ ][ ] | |  |
| 203 | How many people in total have you had sexual intercourse within the past year? | | Give total number:  If 00  Refused to answer | | | | [ ][ ]  99 | | 206 |
| 204 | During the last 12 months, have you ever given someone money or material goods in exchange for sex? | | Yes  No  No response | | | | 1  2  99 | |  |
| 205 | Did you use a condom the last time you had sex? | | Yes  No  No response | | | | 1  2  99 | |  |
| 206 | I don’t want to know the result, but in the past 12 months, *have you* had an HIV test? | | Yes  No  No response | | | | 1  2  99 | |  |
| 207 | In the past 12 months, have you ever drunk an alcohol-containing beverage? For example beer, wine, local brew, local spirit (eg. Gongo) or other alcoholic beverage? | | Yes  No | | | | 1  2 | | 300 |
| 208 | How often do you have a drink containing alcohol? Would you say:  READ RESPONSES: | | 1 -6 times per year  2-4 times a month  2-3 times a week  4 or more times a week | | | | 1  2  3  4 | |  |
| 209 | On average, how many drinks containing alcohol do you have on a typical day when you are drinking? | | 1 or 2  3 or 4  5 or 6  7, 8 or 9  10 or more | | | | 1  2  3  4  5 | |  |
| 210 |  | No | | Once per year | Once per every month | Once per every week | | Daily, almost daily |  |
| a | Do you use six or more drinks on one occasion?  IF YES , READ OUT ANSWERS | 1 | | 2 | 3 | 4 | | 5 |  |
| b | In the past 12 months, have you found that you were not able to stop drinking once you had started?  IF YES , READ OUT ANSWERS | 1 | | 2 | 3 | 4 | | 5 |  |
| c | In the past 12 months ,have you failed to do what was normally expected of you because of drinking?  IF YES , READ OUT ANSWERS | 1 | | 2 | 3 | 4 | | 5 |  |
| d | In the past 12 months, have have you needed a first drink in the morning to get yourself going after a heavy drinking session the previous day?  IF YES , READ OUT ANSWERS | 1 | | 2 | 3 | 4 | | 5 |  |
| e | In the past 12 months, have you had a feeling of guilt or remorse after drinking?  IF YES , READ OUT ANSWERS | 1 | | 2 | 3 | 4 | | 5 |  |
| f | In the past 12 months, have you been unable to remember what happened the night before because of your drinking?  IF YES , READ OUT ANSWERS | 1 | | 2 | 3 | 4 | | 5 |  |
| g | Have you or someone else been injured because of your drinking – either in the past 12 months, or before this? | Yes, during the past 12 months  Yes, but not in the last year  No | | | | | | 1  2  3 |  |
| h | Has a relative, friend, doctor, or other health care worker been concerned about your drinking or suggested you cut down? | Yes, during the past 12 months  Yes, but not in the last year  No | | | | | | 1  2  3 |  |

| SECTION 3: ABOUT ATTITUDES AND SOCIAL NORMS |
| --- |
|  |

In this community and elsewhere, people have different ideas about families and what is acceptable behavior for men and women in the home. We would like to know your views on what is acceptable.

|  | QUESTIONS | CODING CATEGORIES | | | |
| --- | --- | --- | --- | --- | --- |
| 300 | I am going to make a number of statements about men and women in general. When I read the following statements can you please indicate how much you personally agree or disagree.  READ STATEMENTS, THEN ASK IF AGREE OR DISAGREE.  THEN ASK IF AGREE OR STRONGLY AGREE OR DISAGREE OR STRONGLY DISAGREE | In your personal opinion… | | | |
|  |  | I strongly agree | I agree | I disagree | I strongly disagree |
| a | A couple should decide together things that affect the health and well-being of the family | 1 | 2 | 3 | 4 |
| b | It **must** be the man who is the primary provider for the family. | 1 | 2 | 3 | 4 |
| c | Even healthy relationships can include hitting each other as long as the partners love each other. | 1 | 2 | 3 | 4 |
| d | It is perfectly acceptable for women to work outside the home to help support the family economically | 1 | 2 | 3 | 4 |
| e | The leadership of a community should be largely in the hands of men. | 1 | 2 | 3 | 4 |
| f | Sons in the family should be given more encouragement to go to school than daughters | 1 | 2 | 3 | 4 |
| g | It is normal and right that men have more power than woman in the family | 1 | 2 | 3 | 4 |
| h | A man has a good reason to hit his wife if **she** does not complete her household work to his satisfaction | 1 | 2 | 3 | 4 |
| i | A man has a good reason to hit his wife if **she** disobeys him | 1 | 2 | 3 | 4 |
| j | A man has good reason to hit his wife if **she** refuses to have sexual intercourse with him | 1 | 2 | 3 | 4 |
| k | A man does not have any reason to hit his wife in any way | 1 | 2 | 3 | 4 |
| l | A man has a good reason to hit his wife if **she** protests because he has other girlfriends | 1 | 2 | 3 | 4 |
| m | A man has a good reason to hit his wife if **he** suspects that she is unfaithful in marriage | 1 | 2 | 3 | 4 |
| n | A man has a good reason to hit his wife if he finds out that she has been unfaithful in marriage | 1 | 2 | 3 | 4 |

| SECTION 4: ABOUT YOUR RELATIONSHIP |
| --- |

As you know, your partner/wife has been participating in our study for the last 2 years. You might have been with her when she joint this study or you might have met her when she was already part of the study. In this phase of interview, I am going to ask you questions about yourself and about your relationship with the woman who is participating in our study. Please answer all questions regarding your relationship with respect to YOUR CURRENT relationship with the woman participating in our study.

| 400 | How long have you been in a relationship with this woman participating in our study? | Years/Months: ( ) ( ) |  |  |
| --- | --- | --- | --- | --- |
| 401 | Are you currently married or living together with her as if married? | Not married or living together  Married  Living together as if married | 0  1  2 |  |
| 402 | Have you ever been married or lived together with a woman as if married prior to being with this woman? | No  Yes, formerly married  Yes, formerly lived with a woman | 0  1  2 | 404  404 |
| 403 | If the answer is **no,** have you ever been in a relationship with a woman prior to being with this woman? | Yes  No | 1  2 |  |
| 404 | Currently, do you have other wives or do you live with other women as if married? | No, only one  Yes, more than one  No answer | 0  1  99 | 407 |
| 405 | Altogether, how many wives or live-in partners do you have? | Total No:  If never had write 00 |  |  |
| 406 | The woman who is participating in this study (see cover page), is she your first, second, third… wife? | Rank:  If not wife but in a relationship write 99 |  |  |
| 407 | How would you describe your relationship with her? | Very satisfying  Satisfying  Unsatisfying  Very unsatisfying | 1  2  3  4 |  |

When two people marry, live together or are in a relationship, they usually share both good and bad moments. I would now like to ask you some question about your current and past relationships.

|  | QUESTIONS | CODING CATEGORIES |  |  |  |  |  |
| --- | --- | --- | --- | --- | --- | --- | --- |
| 408 | During the last 12 months, did you and your partner discuss the following topics together |  | If happened fill the answer in the shaded area | | | |  |
|  |  | Never | Once | Few times | | Many times | |
| a | … things that happened to you during the day?  Would you say never, once, a few times, or many times? | 1 | 2 | 3 | | 4 | |
| b | … things that happened to herin the day?  Would you say never, once, a few times, or many times? | 1 | 2 | 3 | | 4 | |
| c | … your worries or feelings?  Would you say never, once, a few times, or many times? | 1 | 2 | 3 | | 4 | |
| d | … her worries or feelings?.  Would you say never, once, a few times, or many times? | 1 | 2 | 3 | | 4 | |
| 409 | During the last 12 month, did you ever… |  | If the answer is yes, how often? | | | |  |
| a | … ask your partner for advice to resolve a problem you were facing?  Would you say never, once, a few times, or many times? | 1 | 2 | 3 | 4 | | |
| b | … followed your partner’s advice to resolve a problem you were facing?  Would you say never, once, a few times, or many times? | 1 | 2 | 3 | 4 | | |
| c | … help your partner in finding work?  Would you say never, once, a few times, or many times? | 1 | 2 | 3 | 4 | | |
| d | … encouraged your partner to participate in something outside of the home that was only for *her*benefit?  Would you say never, once, a few times, or many times? | 1 | 2 | 3 | 4 | | |
| e | … made your partner feel appreciated.  Would you say never, once, a few times, or many times? | 1 | 2 | 3 | 4 | | |

| 410 | No matter how well a couple gets along, there are times when they disagree. **In your relationship with your current / mostrecent partner….** |  |  | | |
| --- | --- | --- | --- | --- | --- |
|  |  | Yes | No |  |  |
| a | … would you say that you quarreled in the past 12 months?  (SKIP TO QUESTION 411 IF ANSWER IS NO) | 1 | 2 |  |  |
|  | How often have you quarreled about:  IF HAPPENNED, HAS HAPPENED ONCE, FEW TIMES OR MANY TIMES? | Never | Once | Few times | Many times |
| b | Accusations that you are not fulfilling your responsibilities as a father and husband | 1 | 2 | 3 | 4 |
| c | Her inability or unwillingness to provide for the family | 1 | 2 | 3 | 4 |
| d | Other issues around money and division of resources in the family | 1 | 2 | 3 | 4 |
| e | Her drinking/gambling or drug use | 1 | 2 | 3 | 4 |
| f | Your drinking habit | 1 | 2 | 3 | 4 |
| g | Concerns about outside partners or accusations of infidelity | 1 | 2 | 3 | 4 |
| h | Her refusal to have sex | 1 | 2 | 3 | 4 |
| i | Other issues around sex (frequency, condom use, etc) | 1 | 2 | 3 | 4 |
| j | You disobeying your partner or treating herdisrespectfully. | 1 | 2 | 3 | 4 |
| k | Hertreating you or your children disrespectfully. | 1 | 2 | 3 | 4 |
| l | Youbeing unhappy that shehad taken a loan from a microfinance organization. | 1 | 2 | 3 | 4 |

|  | Who in your family or relationship usually has the final say in how you spend money? |  |  | | |
| --- | --- | --- | --- | --- | --- |
| 411 |  | Yourself | Wife or partner | jointly | Someone else or with someone else |
| a | Food and clothing | 1 | 2 | 3 | 4 |
| b | Health care | 1 | 2 | 3 | 4 |
| c | Large investments such as buying acar, or a house, or a householdappliance | 1 | 2 | 3 | 4 |
| d | Regarding spending time withfamily friends or relatives | 1 | 2 | 3 | 4 |

| SECTION 5: ABOUT YOUR CHILDREN |
| --- |

| 501 | Do you have any BIOLOGICAL children? | Yes  No  If No go to question 504 | 1  2 |
| --- | --- | --- | --- |
| 502 | How many BIOLOGICAL children do you have? | ___ |  |
| 503 | Are they all from your current partner who is participating in this study? | Yes  No | 1  2 |
| 504 | Do you have any non-biological children living in your household? | Yes  No – go to question 600 |  |
| 505 | How many children altogether, who are non-biological to you who live in your household? | ____ |  |
| 506 | Was it you or your partner who wanted to have a  child the last time you had a child or adopted one? | Mostly me  Mostly my partnee  Both equally  Not planned | 1  2  3  4 |
| 507 | Who in your family usually has the final say regarding the health of children at home? | Yourself  Wife/Partner  You and yourwife/partnerjointly  You and someone else jointlySomeone else  Other person………………………………… | 1  2  3  4  5 |

The next questions will ask you about the amount of time you spend with your children doing different things with or for them.

| 508 | Disregarding the help you and/or your partner may get from others, how do/did you and your partnerdistribute the following tasks related to the care of children? | Always me | Usually me | Equally or done together | Usually partner | Always partner | N/A |  |
| --- | --- | --- | --- | --- | --- | --- | --- | --- |
| a | Daily care of child | 1 | 2 | 3 | 4 | 5 | 96 |  |
| b | Staying at home with a child whenhe/she is sick | 0 | 1 | 2 | 3 | 4 | 96 |  |
| c | Collecting child from school/day carecentre | 0 | 1 | 2 | 3 | 4 | 96 |  |
| d | Driving or taking the child to leisuretimeactivities | 0 | 1 | 2 | 3 | 4 | 96 |  |
| e | Playing with the children at home | 0 | 1 | 2 | 3 | 4 | 96 |  |
| f | Talking about personal matters with your children? | 0 | 1 | 2 | 3 | 4 | 96 |  |
| g | Cook or fix food for your children? | 0 | 1 | 2 | 3 | 4 | 96 |  |

| **CHILD DISCIPLINE** | |
| --- | --- |
| 509. Adults use certain ways to teach children the right behavior or to address a behavior problem. I will read various methods that are used. Please tell me if you have used this method with *any child in your household during* the past month.  [A] Took away privileges, forbade something they liked or did not allow them to leave the house.  [B] Explained why their behavior was bad  [C] Shook them.  [D] Shouted, yelled at or screamed at them.  [E] Gave them something else to do.  [F] Spanked, hit or slapped them on the bottom with bare hand.  [G] Hit them on the bottom or elsewhere on the body with something like a belt, hairbrush, stick or other hard object.  [H] Called them dumb, lazy or another name like that.  [I] Hit or slapped them on the face, head or ears.  [J] Hit or slapped them on the hand, arm, or leg.  [K] Beat them up, that is hit him/her over and over as hard as one could. | Yes No  1 2  1 2  1 2  1 2  1 2  1 2  1 2  1 2  1 2  1 2  1 2 |
| 510. Do you believe that in order to bring up, raise, or educate a child properly, the child needs to be physically punished? | Yes 1  No 2  DK / No opinion 8 |

| SECTION 6: ABOUT CHILDHOOD |
| --- |
|  |

In this section I would like to ask you a few questions about things you might have seen or things that might have happened to you when you were a child. Some of these questions might be very difficult to answer. I want you to remember that everything you say here is completely confidential and will not be shared with anyone without your permission.

|  | QUESTIONS | CODING CATEGORIES | | | | | | |  |
| --- | --- | --- | --- | --- | --- | --- | --- | --- | --- |
| 600 | When you were growing up, during the first 15 years of your life . . . |  | | | | | |  |  |
| A | Did your parents/guardians understand your problems and worries? | Yes  No  Don’t know | | | | | | 1  2  96 |  |
| B | Did you feel you were living in a warm and loving household? | Yes  No  Don’t know | | | | | | 1  2  96 |  |
| C | Did you live with a household member who was a problem drinker or used drugs? | Yes  No  Don’t know | | | | | | 1  2  96 |  |
| D | Did you live with a household member whowas depressed, mentally ill or suicidal? | Yes  No  Don’t know | | | | | | 1  2  96 |  |
| E | Did you live with a household member who was sent to jail or prison? | Yes  No  Don’t know | | | | | | 1  2  96 |  |
| F | Were your parents ever separated ordivorced? | Yes  No  Don’t know | | | | | | 1  2  96 |  |
| G | Did your father, mother or guardian die? | Yes  No | | | | | | 1  2 |  |
| H | Did you see or hear a parent or household member in your home being slapped, pushed or beaten with a fist ?  Would you say, never, once, few times, many times? | Never  Once  Few times  Many times | | | | | | 1  2  3  4 |  |
| 601 | These next questions are about certain things YOU YOURSELF may have experienced when you were growing up. During the first 18 years of your life, did a parent, or other adult in the household ever…. | Never | Once | Few times | | | Many times | |  |
| A | Call you bad words, insult you or put you down?  Would you say never, once , few times, or many times. | 1 | 2 | | 3 | 4 | | |  |
| B | Threaten you with physical harm  Would you say never, once or twice, a few times, or many times. | 1 | 2 | | 3 | 4 | | |  |
| C | Spank, slap, kick, punch or beat you up?  Would you say never, once , few times, or many times | 1 | 2 | | 3 | 4 | | |  |
| D | Hit you so hard that you had marks or were injured  Would you say never, once or twice, a few times, or many times. | 1 | 2 | | 3 | 4 | | |  |
| E | Did an adult or person at least 5 years older than you touch or fondle you in a sexual way?  Would you say never, once , few times, or many times | 1 | 2 | | 3 | 4 | | |  |
| F | Make you touch their body in a sexual way?  Would you say never, once , few times, or many times | 1 | 2 | | 3 | 4 | | |  |
| G | Attempt oral, anal, or vaginal intercourse with you?  Would you say never, once , few times, or many times | 1 | 2 | | 3 | 4 | | |  |
| H | Actually have oral, anal, or vaginal intercourse with you?  Would you say never, once , few times, or many times | 1 | 2 | | 3 | 4 | | |  |
|  |  |  |  | |  |  | | |  |
| 602 | This next question is about PHYSICAL FIGHTS. A physical fight occurs when two young people of about the same strength or power choose to fight each other. When you were growing up, during the first 18 years of your life . . . | Never | Once | | Few times | Many times | | |  |
|  | How often were you in a physical fight? | 1 | 2 | | 3 | 4 | | |  |
| 603 | When you were growing up did you see…  READ ANSWERS | Never | Once | | Few times | Many times | | |  |
| A | Someone in your community being beaten up? | 1 | 2 | | 3 | 4 | | |  |
| B | Somebody get stabbed? | 1 | 2 | | 3 | 4 | | |  |
| C | Somebody get shot? | 1 | 2 | | 3 | 4 | | |  |
| D | Somebody threatened with a gun or another weapon? | 1 | 2 | | 3 | 4 | | |  |
| E | Did a thief or burglar forced their way into your house? | 1 | 2 | | 3 | 4 | | |  |

| SECTION 7: ABOUT YOUR COMMUNITY |
| --- |
|  |

I would like to ask you some questions about your life outside your family

|  | QUESTIONS | | CODING CATEGORIES |  |  |
| --- | --- | --- | --- | --- | --- |
| 701 | Who do you spend most of your time with outside work? | Family  Friends  Church/Mosque  Community  Other, please specify _____________ | | | 1  2  3  4  5 |
| 702 | Have you ever been punched, hit or kicked outside the home in the last year? | Yes  No | | | 1  2 |
| 703 | In the past 12 months, how often have you physically fought with other people you are not living with? | Never  Once  Few times  Many times | | | 1  2  3  4 |
| 704 | Have you ever been involved in a fight with a knife, gun or other weapon? | Never  Once  2-3 times  More often | | | 1  2  3  4 |
| 705 | Have you ever been arrested? | Yes  No | | | 1  2 |

I would now like to learn a bit more about the groups and organizations that you are part of in your community.

| 706-707 | Please tell me if you are part of one or several of the following groups. | 706 | | | 707  If **YES for question 706**, please also tell me if you are simply attending, if you are actively contributing or even leading some of the group activities. | | | |  |
| --- | --- | --- | --- | --- | --- | --- | --- | --- | --- |
|  |  | Yes | | No | Member/attends | Active | | Leader |  |
| a. | Religious group | 1 | | 2 | 1 | 2 | | 3 |  |
| b. | Ethnic groups/Cultural groups | 1 | | 2 | 1 | 2 | | 3 |  |
| c. | Economic support groups (non-finance) | 1 | | 2 | 1 | 2 | | 3 |  |
| d. | *Mtaa* based groups | 1 | | 2 | 1 | 2 | | 3 |  |
| e. | Celebration/Burial help groups (vikundivyasherehe/ nakuzikana) | 1 | | 2 | 1 | 2 | | 3 |  |
| f. | Youth group | 1 | | 2 | 1 | 2 | | 3 |  |
| h. | School committee | 1 | | 2 | 1 | 2 | | 3 |  |
| i. | Health committee | 1 | | 2 | 1 | 2 | | 3 |  |
| j. | Sports group | 1 | | 2 | 1 | 2 | | 3 |  |
| k. | Credit/finance group | 1 | | 2 | 1 | 2 | | 3 |  |
| l. | Legal/Professional organization | 1 | | 2 | 1 | 2 | | 3 |  |
| m. | Advocacy groups | 1 | | 2 | 1 | 2 | | 3 |  |
| n. | Others (specify) ___________________ | 1 | 2 | | 1 | 2 | 3 | | |

| 708 | **If someone/an organisation offered training about how to improve relationships between men and women.**  Would you be interesting in participating? | Yes  No | 1  2 | Skip to 710 |
| --- | --- | --- | --- | --- |
| 709 | If yes: what is the main reason for your interest in participating? | Want to improve relationship  Love kids  Want to advance myself  Other___________________ | 1  2  3  4 | Skip to 711  Skip to 711  Skip to 711  Skip to 711 |
| 710 | If no: what is the main reason for not being interested in participating? Not useful, Busy with work, Other? | Not useful  Busy with work  No time  Men do not do this  Other ____________________ | 1  2  3  4  5 |  |

Now I am going to ask you some questions about how the community functions and deals with problems.

| 711 | Neighbors in this community tend to know each other well  PROBE:Do you agree or disagree with this statement? Do you strongly agree or strongly disagree? | Strongly agree  Agree  Disagree  Strongly disagree | 1  2  3  4 |
| --- | --- | --- | --- |
| 712 | In this community, people, generally trust each other in matters of lending and borrowing  PROBE:Do you agree or disagree with this statement? Do you strongly agree or strongly disagree? | Strongly agree  Agree  Disagree  Strongly disagree | 1  2  3  4 |
| 713 | In this area, it is safe to walk around at night  PROBE:Do you agree or disagree with this statement? Do you strongly agree or strongly disagree? | Strongly agree  Agree  Disagree  Strongly disagree | 1  2  3  4 |
| 714 | Suppose two people in this village/neighborhood had a serious dispute with each other. Who do you think would primarily help resolve the dispute? | No one; people work it out between themselves  Family/household members  Neighbours  Groups’ members  Community leaders  Religious leaders  Judicial leaders  Other (specify) . . . . . . . . . . . . . | 1  2  3  4  5  6  7  8 |
| 715 | How comfortable do you feel about asking for advice from your neighbour or friend? Would you say: (READ RESPONSES) | Very confident  Confident but would need to be encouraged  Not confident at all  Don’t know | 1  2  3  96 |
| 716 | Neighbours often have problems (eg, around raising children, relationships or finding jobs). How confident do you feel about offering advice to your neighbour or friend? Would you say: (READ RESPONSES) | Very confident  Confident but would need to be encouraged  Not confident at all  Don’t know | 1  2  3  96 |

**WHEN YOU ARE DONE**

| **Interview details – END**  Date of interview:  Time interview ended:  Name of interviewer:  Are you the same interviewer as at the beginning? No **Yes**  Comments: |
| --- |

| **Interview closure**  I would like to thank you very much for helping us. I appreciate the time that you have taken. I realize that these questions my have been difficult to answer, but it is only by hearing from men themselves that we can really understand about relationships and experiences in life. |
| --- |
