# Supplementary material for: Couples data from north-western Tanzania: Insights from a survey of male partners of women enrolled in the MAISHA cluster randomized trial of an intimate partner violence prevention intervention
Source: PLoS One. 2020 Oct 2;15(10):e0240112. doi: 10.1371/journal.pone.0240112 (PMC7531846; doi:10.1371/journal.pone.0240112)
Supplement: S4 Questionnaire — (PDF) [file pone.0240112.s004.pdf]

HOJAJI YA WASHIRIKIWENZA WA KIUME

HOJAJI HII NI SIRI IKISHAJAZWA

SEHEMU YA 1: KUHUSU WEWE 3

SEHEMU YA 2: KUHUSU WEWE NA AFYA YAKO 6

SEHEMU YA 3: KUHUSU MITAZAMO NA KANUNI ZA JAMII 8

SEHEMU YA 4: KUHUSU UHUSIANO WAKO 9

SEHEMU YA 5: KUHUSU WATOTO WAKO 12

SEHEMU YA 6: KUHUSU UTOTONI 13

SEHEMU YA 7: KUHUSU JAMII YAKO 15

Utambulisho

Utangulizi

Habari, jina langu ni_____________________, Ninatokea katika Taasisi ya taifa ya utafiti wa magonjwa ya binadamu (NIMR) kituo cha Mwanza na kitengo cha tafiti za kuzuia magonjwa ya binadamu (MITU) cha Mwanza. Kama unavyofahamu, wewe umekubali kushiriki katika ufafiti ambao tunaufanya kwa sasa hapa Mwanza. Kama sehemu ya utafiti huu tungependa kukuuliza maswali kadhaa kukuhusu wewe binafsi, kaya yako, mahusiano yako na jamii yako. Baadhi ya maswali ni nyeti, lakini kila kitu utakachotuambia kitatunzwa kwa usiri na hawatashirikishwa watu wengine. Ninapenda kukusisitiza uwe mkweli, maana hakuna majibu yaliyo sahihi au yasiyokuwa sahihi. Unaweza kukatisha mahojiano wakati wowote, au unaweza ukaamua kutokujibu swali. Hii haitaathiri ushiriki wako katika utafiti huu hapo baadaye. Kama utagundua kitu chochote kinacholeta maudhi kati ya mambo tuliyojadili, na ungependa kumweleza mtu mwingine hapo baadaye, tunaweza kukusaidia kwa hili.

Mahojiano haya yatachukua masaa mawili au zaidi kumalizika. Kwa hili, itakuwa bora zaidi kama tutakuwa sehemu ambayo mahojiano yetu hayatakatishwa na ambayo haina bughudha. Je, hapa ni mahali pazuri kufanyia mazungumzo au tunaweza kwenda mahali pengine ambapo tunaweza kuongea kwa faragha?

Je, una maswali yoyote?

KABLA YA KUANZA

**Namba ya Utambulisho ya Mshiriki**	
**Namba ya Mshiriki wa Kike**	**G [] [] [] [] - [] [] [] [] - [] []**
**Namba ya Mshiriki wa Kiume**	**G [] [] [] [] - [] [] [] [] - [] []**

TAFADHALI TUMIA NAMBA YA UTAMBULISHO YA MSHIRIKI WA KIKE PAMOJA NA ORODHA YA UTAMBULISHO ILI KUMTAMBUA MWANAMKE HUSIKA. KISHA THIBITISHA KUWA MWANAUME UNAYEMHOJI ANA UHUSIANO NAMWANAMKE HUYU.

**Ndiyo**	**Hapana**
Yupo katika uhusiano na mwanamke huyu (namba ya utambulisho ya mshiriki wa kike).<br>Endelea na hojaji.	Hayupo katika uhusiano na mwanamke huyu (namba ya utambulisho ya mshiriki wa kike).<br>Sitisha mahojiano na mjulishe kiongozi wa timu.

! TAFADHALI JAZA NAMBA KAMILI YA UTAMBULISHO YA MSHIRIKI CHINI YAKILAUKURASA WA HOJAJI HII!

Maelezo Kuhusu Mahojiano - MWANZO

Tarehe ya mahojiano:**[] [] / [] [] [] / [] [] [] [] tarehe/ mwezi/ mwaka**

Muda wa kuanza mahojiano:**[] []: [] []**

Jina la mhojaji:**[] [] []**

SEHEMU YA 1: KUHUSU WEWE

Sasa ningependa kuuliza maswali machache kuhusu wewe mwenyewe, aina ya kazi unayofanya na jinsi unavyojisikia au ulivyojisikia kuhusu baadhi ya mambo ambayo yamekutokea.

	MASWALI	AINA ZA MISIMBO
100	Una miaka mingapi?<br><br>MHOJIWA AKADIRIE KAMA HAJUI UMRI HALISI	Miaka: [] []
101	Ni kwa muda gani umekuwa ukiishi Mwanza?	Miaka( ) ( ) Miezi ( ) ( )
102	Ulizaliwa tarehe ngapi?<br><br>(NAKILI TAARIFA NYINGI IWEZEKANAVYO. JAZA 96 KAMA HAFAHAMU SIKU, 969 KAMA HAFAHAMU MWEZI, NA 9696 KAMA HAFAHAMU MWAKA)	SIKU [ ][ ]<br><br>MWEZI [ ][ ][ ]<br><br>MWAKA [ ][ ] [ ][ ]
102	Wewe ni raia wa Tanzania?	Mtanzania 1<br>Si Mtanzania 2
103	Umekulia wapi?<br><br>DODOSA KAMA INAHITAJIKA: Kabla haujatimiza umri wa miaka 12, uliishi wapi kwa muda mrefu zaidi?	Jamii hii /mtaa huu 1<br><br>Sehemu nyingine kijijini hapa Tanzania 2<br><br>Mji/jiji jingine hapa Tanzania 3<br><br>Nchi nyingine 4<br><br>Sijui/sikumbuki 96<br><br>Amekataa kujibu/hakuna jibu 99
104	Nani amekulea	Baba na mama 1<br>Mama kwa kiasi kikubwa 2<br>Baba kwa kiasi kikubwa 3<br>Mtu mwingine.Tafadhali mtaje:__________ 4
105	Ni nani aliyekulea?	Baba na mama 1<br>Mama kwa kiasi kikubwa 2<br>Baba kwa kiasi kikubwa 3<br>Mtu mwingine.Tafadhali mtaje:__________ 4
106	Unaweza kueleza wewe ni muumini wa dini gani?	Muislamu 1<br><br>Msabato 2<br><br>Tanzania Assemblies of God (TAG) 3<br><br>Mkatoliki 4<br><br>Mlutheri 5<br><br>Moravian 6<br><br>Pentekoste 7<br><br>African Inland Church (AIC) 8<br><br>Hana dini 9<br><br>Dhehebu jingine (Taja): 10
107	Wewe ni kabila gani?	Msukuma 1<br>Mjita 2<br>Mzinza 3<br>Mnyiramba 4<br>Mkara/ Mkerewe 5

		Mhaya	6
		Mjaluo	7
		Mkuria/ Mshashi	8
		Mchaga	9
		Mhindi	10
		Mwarabu	11
		Kabila jingine (Taja):	12
107	Umemaliza kiwango gani cha juu cha elimu?	Sijaenda shule kabisa	1
		Sijamaliza shule ya msingi	2
		Nimemaliza shule ya msingi	3
		Sijamaliza shule ya sekondari	4
		Sekondari (Kidato cha 1-IV)	5
		Sekondari (Kidato cha V-VI)	6
		Mafunzo ya chuo mara baada ya shule ya msingi/sekondari na kabla ya chuo kikuu	7
		Chuo kikuu	8

Sasa ningependa kufahamu zaidi juu ya njia unazozitumia kujipatia pesa.

108	Je, chanzo kikuu cha mapato yako na familia yako ni kipi?<br><br>INARUHUSU MAJIBU ZAIDI YA MOJA	Ndiyo	Hapana	
a	Pesa kutokana na shughuli binafsi	1	2	
b	Msaada kutoka kwa mke/mwenzi	1	2	
c	Msaada kutoka kwa ndugu wengine	1	2	
d	Malipo baada ya kustaafu	1	2	
e	Huduma/ustawi wa jamii	1	2	
f	Nyingine (Taja)	....		
109	Je, umewahi kujipatia pesa yako mwenyewe katika kipindi cha miezi 12 iliyopita?	Ndiyo 1<br>Hapana 2		220
110	Je, umejiajiri wewe mwenyewe au umeajiriwa na mtu mwingine/shirika?	Nimejiajiri 1<br>Ninafanya kazi kwa mtu au shirika 2<br>Yote ni kweli 3		
111	Je, kazi hii ni ya kudumu au unaifanya mara mojamoja?	Mara kwa mara 1<br>Mara moja tu 2		
	Kama umejiajiri…..			
112	Siku yako ya kawaida ya kazi, unafanya kazi masaa mangapi?<br>ANDIKA 96 KAMA HAJUI	Masaa: [] []		
113	Katika siku/ wiki/ mwezi wa kawaida kazi hii inakuingizia kipato gani kwa siku/ wiki/ mwezi?<br>JAZA KWA SIKU, WIKI AU MWEZI	Siku: [] [] [] [] [] []<br>Wiki: [] [] [] [] [] []<br>Mwezi: [] [] [] [] [] []		

114	Siku hizi, familia nyingi zina wakati mgumu katika kufikia malengo. Ningependa kujifunza zaidi jinsi familia yako inavyojimudu.<br><br>SISITIZA KWAMBA UNAZUNGUMZIA KUHUSU MIEZI 12 ILIYOPITA<br><br>Ndani ya miezi 12 iliyopita…	Hata kidogo	Kama jibusiyo **HATA KIDOGO**…<br>Mara moja	Mara chache	Mara nyingi
a	…ulikuwa na wasiwasi mkubwa juu ya hali yako ya kifedha kwa ujumla?<br>Imewahi kutokea au haijawahi kutokea?	1	2	3	4
b	…umepata tatizo la kununua chakula au mahitaji mengine muhimu ya familia yako?<br>Imewahi kutokea au haijawahi kutokea?	1	2	3	4
c	…ulilazimika kukopa pesa ili kulipa kodi ya nyumba au bili nyingine?<br>Imewahi kutokea au haijawahi kutokea?	1	2	3	4
d	…mmojawapo wa wanafamilia alihitaji kuonana na daktari lakini hakuweza kwa sababu haukuwa na pesa ya kutosha?<br>Imewahi kutokea au haijawahi kutokea?	1	2	3	4
e	…watoto wako walikosa kwenda shule kwa sababu haukuwa na pesa ya ada, sare za shule au mahitaji?<br>Imewahi kutokea au haijawahi kutokea?	1	2	3	4
f	…ilitokea wewe au mtoto wako yeyote hakula kitu chochote siku nzima kwa sababu hakukuwa na chakula cha kutosha?<br>Imewahi kutokea au haijawahi kutokea?	1	2	3	4

Ushiriki wa mwenza katika mikopo midogomidogo

**115**	Ndani ya miezi 12 iliyopita, je, mwenza wako amewahi kuchukua mkopo wa fedha kutoka kwenye mashirika/asasi yoyote inayotoa mikopo midogomidogo tofauti na BRAC? Kama vile SACCOS,, FINCA, PRIDE au asasi nyingine?	Ndiyo 1<br>Hapana 2	116<br>200

**116**	Je ni kwa kiasi gani unaamini kuwa mwenza wako ana uwezo wa kulipa deni hilo?Je, unaweza kusema kwamba: Ninajiamini sana, Ninajiamini kiasi fulani, Sijiamini sana?	Ninajiamini sana 1<br>Ninajiamini kiasi fulani 2<br>Sijiamini sana 3	
**117**	Fedha anazopata mwenza wako kutokana na mikopo midogomidogo ni muhimu kiasi gani kwa familia?<br>Je, ni muhimu sana, ni muhimu kiasi, au si muhimu hata kidogo?	Muhimu sana 1<br>Muhimu kiasi 2<br>Si muhimu hata kidogo 3	
**118**	Ndani ya miezi 12 iliyopita, kwa ujumla, ni kwa jinsi gani mwenza wako kuwa mwanachama wa asasi au vikundi vingine vya mikopo midogomidogo kumeathiri uhusiano wako na mwenzi wako? Unaweza kusema kwamba: hakujaathiri uhusiano na mwenzi wako, kumefanya uhusiano wako uwe na matatizo zaidi, au kumeboresha uhusiano wako?	Hakujaathiri uhusiano 1<br>Uhusiano umekuwa magumu zaidi 2<br>Kumeboresha uhusiano 3	

**SEHEMU YA 2: KUHUSU WEWE NA AFYA YAKO**

Sasa ningependa kukuuliza maswali kadhaa kuhusu afya yako.

200	Katika kipindi cha wiki 4 zilizopita, umesumbuliwa na tatizo lolote kati ya haya yafuatayo?	Ndiyo	Hapana
a	Je, mara kwa mara huwa unaumwa kichwa?	1	2
b	Je, hamu yako ya kula huwa sio nzuri?	1	2
c	Je, huwa unalala vibaya? Kama vile kukosa usingizi, kuamka usiku wa manane zaidi ya mara tatu, au kuamka asubuhi sana kisha ukakosa usingizi?	1	2
d	Je, huwa unapata hofu kirahisi?	1	2
e	Je, mikono yako huwa inatetemeka?	1	2
f	Je, huwa unasikia uoga au wasiwasi?	1	2
g	Je, mfumo wa kusaga chakula mwilini mwako huwa sio mzuri? Kama vile kutopata choo mara kwa mara, unasikia kichefuchefu au hauna hamu ya kula.	1	2
h	Je, huwa unapata shida kufikiria kwa umakini?	1	2
i	Je, huwa unajisikia hunaa na furaha?	1	2
j	Je, huwa unalia kuliko kawaida? Kwa mfano, unalia kila siku au zaidi ya mara moja kwa siku, kwa sababu ya matatizo?	1	2
k	Je, huwa unaona vigumu kufurahia shughuli zako za kila siku?	1	2
l	Je, huwa unaona ni vigumu kufanya maamuzi yoyote?	1	2
m	Je, shughuli zako za kila siku haziendi vizuri?	1	2
n	Je, huwa unashindwa kutoa mchango au kufanya mambo muhimu katika maisha?	1	2
o	Je, umepoteza hamu ya kujihusisha na mambo mbalimbali?	1	2
p	Je, huwa unajisikia kuwa mtu asiyekuwa na thamani?	1	2
q	Je, mawazo ya kujiua yamekuwa yakikujia akilini?	1	2
r	Je, huwa unajisikia vibaya tumboni?	1	2
s	Je, huwa unachoka kirahisi?	1	2

Sasa ningependa kukuuliza maswali kadhaa kuhusumasuala ya kujamiiana.Baadhi ya maswali yanaweza kuwa nyeti na ya aibu kuyajibu. Tafadhali kumbuka kwamba taarifa hii ni siri na unaweza kuamua kutoyajibu maswali haya.

201	Ulianza kujamiiana ukiwa na umri gani?	Umri katika miaka: [ ][ ]<br>99 kama hajawahi kujamiiana kamwe	207

202	Maishani mwako umewahi kujamiiana na watu wangapi jumla?	Andika idadi ya jumla: [] []	
203	Kwa mwaka mmoja uliopita, umejamiiana na watu wangapi jumla?	Andika idadi ya jumla: [] []<br>Kama ni 00<br>Amekataa kujibu 99	225
204	Katika miezi 12 iliyopita, umewahi kumpa mtu pesa au mali nyingine ili uweze kujamiiana naye?	Ndiyo 1<br>Hapana 2<br>Hakuna jibu 99	
205	Je, mara ya mwisho ulipojamiiana ulitumia kondomu?	Ndiyo 1<br>Hapana 2<br>Hakuna jibu 99	
206	Sitaki kujua majibu yalikuwaje, lakini katika miezi 12 iliyopita,*umewahi* kupima VVU?	Ndiyo 1<br>Hapana 2<br>Hakuna jibu 99	
**207**	Katika miezi 12 iliyopita, umewahi kunywa kinywaji chenye kilevi? Kwa mfano bia, mvinyo, pombe za kienyeji, gongo au pombe za aina nyingine?	Ndiyo 1<br>Hapana 2	300
**208**	Ni mara ngapi unakunywa kinywaji chenye kilevi? Je, unaweza kusema ni:<br>SOMA MAJIBU:	Mara 1-6 kwa mwaka 1<br>Mara 2-4 kwa mwezi 2<br>Mara 2-3 kwa wiki 3<br>Mara 4 au zaidi kwa wiki 4	
**209**	Kwa wastani, huwa unatumia vivywaji vingapi vyenye kilevi katika siku ya kawaida unapokunywa?	1 au 2 1<br>3 au 4 2<br>5 au 6 3<br>7, 8 au 9 4<br>10 au zaidi 5	

210		Hapana	Mara moja kwa mwaka	Mara moja kwa kila	Mara moja kwa kila wiki	Kila siku, karibu kila siku
a	Huwa unatumia vinywaji sita au zaidi katika tukio moja??<br>KAMA JIBU NI NDIYO, SOMA MAJIBU	1	2	3	4	5
b	Ndani ya miezi 12 iliyopita uligundua kwamba ilikuwa vigumu kwako kuacha kunywa pombe mara ulipoanza kunywa?KAMA JIBU NI NDIYO, SOMA MAJIBU	1	2	3	4	5
c	Ndani ya miezi 12 iliyopita ulishindwa kufanya kile ambacho kwa kawaida kilitegemewa kutoka kwako kwa sababu ulikunywa pombe?<br>KAMA JIBU NI NDIYO, SOMA MAJIBU	1	2	3	4	5
d	Ndani ya miezi 12 iliyopita ulihitaji kuzimua (kunywa kinywaji chenye kilevi kwanza asubuhi) ili uweze kuendelea na shughuli zako baada ya kuwa umekunywa pombe nyingi siku iliyopita<br>KAMA JIBU NI NDIYO, SOMA MAJIBU	1	2	3	4	5
e	Ndani ya miezi 12 iliyopita ulijihisi kuwa na hatia au ulijuta baada ya kunywa pombeKAMA JIBU NI NDIYO, SOMA MAJIBU	1	2	3	4	5
f	Katika miezi 12 iliyopita, umekuwa huwezi kukumbuka kilichotokea usiku uliopita kwa sababu ya kunywa?<br>KAMA JIBU NI NDIYO, SOMA MAJIBU	1	2	3	4	5
g	Je, wewe au mtu mwingine amewahi kujeruhiwa kwa sababu ya unywaji wako wa pombe? – iwe ndani ya miezi 12 iliyopita au kabla ya hapo?	Ndiyo, kwa miezi 12 iliyopita 1<br>Ndiyo, sikwa miezi 12 iliyopita 2<br>Hapana 3				
h	Je, jamaa, marafiki, daktari au watoa huduma wengine wa afya wamekuwa na wasiwasi juu ya unywaji wako wa pombe au wamekushauri upunguze?	Ndiyo, kwa miezi 12 iliyopita 1<br>Ndiyo, si kwa miezi 12 iliyopita 2<br>Hapana 3				

SEHEMU YA 3: KUHUSU MITAZAMO NA MILA/DESTURI ZA KIJAMII

Katika jamii hii na kwingineko, watu wana maoni tofauti kuhusu familia na tabia zipi zinakubalika na zipi hazikubaliki kwa wanaume na wanawake nyumbani. Tungependa kujua maoni yako kuhusu nini kinakubalika.

	MASWALI	AINA ZA MISIMBO			
300	Nitatoa kauli mbalimbali kuhusu wanaume na wanawake kwa jumla. Ninaposoma kauli zifuatazo, tafadhali unaweza kuonyesha ni kwa kiasi gani wewe binafsi unakubaliana nazo au hukubaliani nazo. Kisha, tafadhali onyesha ni kwa kiasi gani unafikiri marafiki zako na majirani zako watakubaliana au hawatakukubaliana na kauli hizo.<br><br>SOMA KAULI, KISHA ULIZA KAMA ANAKUBALIANA AU HAKUBALIANI NAZO.<br>KISHA ULIZA KAMA ANAKUBALIANA AU ANAKUBALIANA SANA AU HAKUBALIANI AU HAKUBALIANI HATA KIDOGO	Kwa maoni yako binafsi...			
		Ninakubaliana sana	Ninakubaliana	Sikubaliani	Sikubaliani hata kidogo
a	Wanandoa wanapaswa kuamua pamoja mambo yanayohusu afya na ustawi wa familia.	1	2	3	4
b	Mwanaume**ndiye**lazima awe mtafutaji mkuu katika familia.	1	2	3	4
c	Hata mahusiano yaliyo mazuri yanaweza kujumuisha kupigana ili mradi tu wenzi wanapendana	1	2	3	4
d	Inakubalika kabisa wanawake kufanya kazi nje ya nyumbani ili kusaidia familia kiuchumi.	1	2	3	4
e	Uongozi wa jamii kwa kiasi kikubwa unatakiwa uwe mikononi mwa wanaume.	1	2	3	4
f	Watoto wa kiume katika familia wanapaswa kupewa motisha zaidi kwenda shule kuliko wa kike.	1	2	3	4
g	Ni jambo la kawaida na sahihi kwa mwanaume kuwa na mamlaka zaidi kuliko mwanamke katika familia.	1	2	3	4
h	Mwanaume ana sababu nzuri ya kumpiga mke wake kama hajakamilisha kazi za nyumbani kwa kiasi kinachoridhisha.	1	2	3	4
i	Mwanaume ana sababu nzuri ya kumpiga mkewe kamamkewehamheshimu.	1	2	3	4
j	Mwanaume ana sababu nzuuriya kumpiga mkewe kamamkewe iwapo aatakataa kujamiiana naye.	1	2	3	4
k	Mwanaume hana sababu yoyote ile i ya kumpiga mkewe katika hali yoyote ile	1	2	3	4
l	Mwanaume ana sabaabu nzuri ya kumpiga mkewe kamamkeweatapinga mumewe kuwa na wapenzi wengine.	1	2	3	4
m	Mwanaume ana sababu nzuri ya kumpiga mkewe kamaatahisi kuwa mkewe si mwaminifu katika ndoa.	1	2	3	4
n	Mwanaume ana sababu nzuri ya kumpiga mkewe akigundua kuwa mkewe amekuwa si mwaminifu katika ndoa.	1	2	3	4

SEHEMU YA 4: KUHUSU UHUSIANO WAKO

Kama unavyojua, mwenza/ mke wako amekuwa akishiriki katika utafiti wetu kwa miaka miwili iliyopita. Inawezekana wakati alipojiunga na utafiti huu tayari ulikuwa naye au ulikutana naye wakati alipokuwa ameshajiunga na utafiti huu. Katika hojaji hii, nitakuuliza maswali kuhusu wewe mwenyewe na kuhusu uhusiano wako na mwanamke huyu anayeshiriki katika utafiti wetu. Tafadhali jibu maswali yote kuhusu uhusiano wako kwa kuzungumzia UHUSIANO ULIO NAO SASA na mwanamke huyu anayeshiriki katika utafiti wetu.

400	Ni kwa muda gani umekuwa katika uhusiano na mwanamke huyu anayeshiriki katika utafiti wetu?	Miaka: Miezi: [ ] [ ]		
401	Je, kwa sasa umeoana naye au unaishi naye kinyumba?	Hatujaoana wala hatuishi pamoja<br>Tumeoana<br>Tunaishi wote kaama wanandoa	0<br>1<br>2	
402	Je, umemuoa au mnaishi pamoja kama mke na mume?	Hapana<br>Ndiyo, nimewahi kuoa<br>Ndiyo, nimewahi kuishi na mwanamke	0<br>1<br>2	 404<br>404
403	Kama**jibu ni hapana,**umeshawahi kuwa na mahusiano na mwanamke mwingine kabla ya kuwa na mwanamke huyu?	Ndiyo<br>Hapana	1<br>2	
404	Kwa sasa una wake wengine au unaishi na wanawake wengine kama wanandoa?	Hapana, mmoja tu<br>Ndiyo, zaidi ya mmoja<br>Hakuna jibu	0<br>1<br>99	407
405	Kwa jumla, una wanawake wangapi uliooa au unaoishi nao nyumba moja? ?	Idadi ya jumla:		
406	Mwanamke anayeshiriki katika utafiti huu (tazama jalada) ni mke wako ya kwanza, wa pili, wa tatu...?	Kiwango:		
407	Unauelezeaje uhusiano wako na yeye?	Unaridhisha sana<br>Unaridhisha<br>Hauridhishi<br>Hauridhishi kabisa	0<br>1<br>2<br>3	

Wakati watu wanapooana, au wanapoishi pamoja au wanapokuwa katika uhusiano, kwa kawaida hushirikiana katika shida na raha. Sasa ningependa kukuuliza maswali kadhaa kuhusu mahusiano yako ya sasa na ya zamani.

	MASWALI	AINA ZA MISIMBO			
**408**	Katika miezi 12 iliyopita, wewe na mwenza wako mlijadili pamoja mambo yafuatayo:		Kama mlijadili, jaza jibu katika eneo lenye kivuli.		
		Hata kidogo	Mara moja	Mara chache	Mara nyingi
a	… mambo yaliyokutokea katika siku?<br>Unaweza kusema haijawahi kutokea, mara moja, mara chache au mara nyingi?	1	2	3	4
b	… mambo yaliyomtokea yeye katika siku?<br>Unaweza kusema haijawahi kutokea, mara moja, mara chache au mara nyingi?	1	2	3	4
c	… wasiwasi au hisia ulizokua nazo?<br>Unaweza kusema haijawahi kutokea, mara moja, mara chache au mara nyingi?	1	2	3	4
d	… wasiwasi au hisia zake?<br>Je, unasema haikutokea hata kidogo, au ilitokea mara moja, au mara chache, au mara nyingi?	1	2	3	4

409	Katika miezi 12 iliyopita, je, umewahi ...		Kama jibu ni ndiyo; je, ni mara ngapi?		
a	... kumuomba mwenza wako ushauri ili kutatua tatizo lililokukabili?<br>Je, unasema haikutokea hata kidogo, au ilitokea mara moja, au mara chache, au mara nyingi?	1	2	3	4
b	... kufuata ushauri wa mwenza wako ili kutatua tatizo lililokukabili?<br>Je, unasema haikutokea hata kidogo, au ilitokea mara moja, au mara chache, au mara nyingi?	1	2	3	4
c	... kumsaidia mwenza wako kutafuta kazi?<br>Je, unasema haikutokea hata kidogo, au ilitokea mara moja, au mara chache, au mara nyingi?	1	2	3	4
d	... kumhimiza mwenza wako kushiriki jambo nje ya nyumbani ambalo lilikuwa ni kwafaida *yake tu?*<br>Je, unasema haikutokea hata kidogo, au ilitokea mara moja, au mara chache, au mara nyingi?	1	2	3	4
e	... kumfanya mpenzi wako ajisikie anathaminiwa?<br>Je, unasema haikutokea hata kidogo, au ilitokea mara moja, au mara chache, au mara nyingi?	1	2	3	4

410	Haijalishi wanandoa wanaelewana kiasi gani, kuna wakati huwa hawakubaliani. Katika uhusiano wako na mwenzi wako wa sasa au wa hivi karibuni....	Ndiyo	Hapana		
a	... unaweza kusema kwamba mlizozana katika miezi 12 iliyopita?<br>(RUKIA swali namba 607 kama jibu ni hapana)	1	2		
	Ni mara ngapi mmezozana kuhusu:<br>KAMA ILITOKEA, ILIKUWA NI MARA MOJA, MARA CHACHE AU MARA NYINGI?	Hata kidogo	Mara moja	Mara chache	Mara nyingi
b	Shutuma kuwa hutimizi wajibu wako kama baba na mume	1	2	3	4
c	Mwenzi wako kutokuwa na uwezo au nia ya kutimiza mahitaji ya familia.	1	2	3	4
d	Masuala mengine yanayohusiana na fedha na mgawanyo wa rasilimali katika familia	1	2	3	4
e	Tabia ya mwenzi wako ya kunywa/kucheza kamari au kutumia madawa ya kulevya	1	2	3	4
f	Tabia yako ya kunywa pombe	1	2	3	4
g	Wasiwasi kuhusu wenzi wa nje au tuhuma za kutokuwa mwaminifu katika ndoa/mahusiano.	1	2	3	4
h	Mwenzi wako kukataa kufanya mapenzi	1	2	3	4
i	Masuala mengine yanayohusiana na kufanya mapenzi (idadi ya kufanya mapenzi, matumizi ya kondomu, n.k.)	1	2	3	4
j	Wewe kutomtii mwenza wako au kumkosea heshima	1	2	3	4
k	Mwezi wako kukukosea heshima wewe au watoto wako	1	2	3	4
l	Wewe kutofurahishwa na kitendo cha mwenzi wako cha kuchukua mkopo kutoka taasisi ndogo inayotoa mikopo	1	2	3	4

411. Kwa kawaida ni nani katika familia yenu au katika uhusiano wenu ana kauli ya mwisho kuhusu mnavyotumia pesa kwa ajili ya...	Wewe	Mke au mwenza	Nyinyi nyote	Mtu mwingine au na mtu mwingine
a Chakula na mavazi	1	2	3	4
b Huduma za afya	1	2	3	4
c Uwekezaji mkubwa kama vile kununua gari, au nyumba, au vyombovya ndani	1	2	3	4
d Kuwa pamoja na marafiki wa familia au ndugu	1	2	3	4

SEHEMU YA 5: KUHUSU WATOTO WAKO

501	Je, una watoto wowote wa KUZAA MWENYEWE?	Ndiyo 1<br>Hapana 2
502	Una watoto wangapi wa KUZAA MWENYEWE?	___
503	Je, wote umewazaa na mwenza wako wa sasa?	Ndiyo 1<br>Hapana 2
504	Kwa jumla, kuna watoto wangapi (wa kuzaa mwenyewe na wasio wa kuzaa mwenyewe) wanaoishi katika kaya yako?	____
505	Kwa kawaida ni nani katika familia yenu ana kauli ya mwisho kuhusu afya ya watoto nyumbani?	Wewe 1<br>Mke wako/ Mwenza wako 2<br>Wewe na mke/mwenza pamoja 3<br>Mtu mwingine 4<br>Wewe na mtu mwingine pamoja 5<br>Mtu mwingine: 6

Maswali yafuatayo yatakuuliza kuhusu kiasi cha muda unaotumia kufanya mambo mbalimbali na watoto wako au kwa ajili yao.

506	Ukiacha msaada ambao wewe na/au mwenza wako mliupata kutoka kwa watu wengine, je, wewe na mwenza wako mnagawanaje/ mligawanaje kazi zifuatazo za kulea watoto?	Wakati wote ni mimi	Kwa kawaida ni mimi	Tuligawana kwa usawa au tulifanya [illegible]	Kwa kawaida ni mwenza wangu	Wakati wote ni mwenza	Haihusiki
a.	Kulea watoto kila siku	0	1	2	3	4	96
b.	Kukaa nyumbani na watoto wakati wewe/ yeye anapokuwa mgonjwa	0	1	2	3	4	96
c.	Kuwachukua watoto kutoka shuleni/ kituo cha kulelea watoto	0	1	2	3	4	96
d.	Kuwapeleka watoto matembezini	0	1	2	3	4	96
e.	Kucheza na watoto nyumbani	0	1	2	3	4	96
f	Kuzungumzia mambo binafsi na watoto wenu	0	1	2	3	4	96
g	Kupika au kuandaa chakula cha watoto wenu	0	1	2	3	4	96

NIDHAMU YA WATOTO

507 Watu wazima hutumia njia fulanifulani kuwafundisha watoto tabia njema au kuwakanya.Nitasoma kwa sauti njia mbalimbali zinazotumika. Tafadhali niambie iwapoulitumia njia ifuatayo kwa*mtoto yeyote katika kaya yako*katika kipindi cha mwezi mmoja uliopita.

	NDIYO	HAPANA
[A] Nilimnyima upendeleo, nilimzuia kitu fulani anachokipendeleaau sikumruhusu kuondoka nyumbani.	1	2
[B] Nilimweleza kwa nini tabia yake ilikuwa mbaya.	1	2
[C] Nilimtikisatikisa.	1	2
[D] Nilimkaripia kwa sauti kali.	1	2
[E] Nilimpa kazi nyingine ya kufanya.	1	2
[F] Nilimchapa makofi matakoni.	1	2
[G] Nilimchapa matakoni au sehemu nyingine za mwili na kitu kama mkanda, chanuo, fimbo au kitu kingine kigumu.	1	2
[H] Nilimwambia kuwa ni mpumbavu, mvivu au jina jingine kama hilo.	1	2
[I] Nilimpiga usoni, kichwani au masikioni.	1	2
[J] Nilimpiga mikononi au miguuni.	1	2
[K] Nilimchapa kwelikweli.	1	2

**UCD3.Je, unaamini kwamba ili kumlea au kumwelimisha mtoto vizuri, mtoto anahitaji kupewa adhabu inayoumiza mwili?**	NDIYO 1<br>HAPANA 2<br>SIJUI/ SINA MAONI 8

SEHEMU YA 6: KUHUSU KIPINDI CHA UTOTO WAKO

Katika sehemu hii ningependa nikuulize maswali machache kuhusu mambo ambayo inawezekana umeyaona au yalitokea kwako ulipokuwa mtoto. Baadhi ya maswali haya yanaweza kuwa magumu sana kuyajibu. Naomba ukumbuke kwamba kila kitu utakachokisema hapa ni siri na hatashirikishwa mtu mwingine yeyote bila ruhusa yako

	MASWALI	AINA ZA MISIMBO
600	Wakati unakua, kuanzia ututoni hadi umri wa miaka 15...	
a	Je wazazi/walezi wako walifahamu kuhusu matatizo na hofu zako?	Ndiyo 1<br>Hapana 2<br>Sijui 96
b	Je ulihisi kuwa ulikuwa unaishi katika kaya yenye upendo?	Ndiyo 1<br>Hapana 2<br>Sijui 96
c	Uliishi na mmojawapo wa wanafamilia ambaye alikuwa na matatizo ya ulevi wa pombe au kutumia madawa ya kulevya?	Ndiyo 1<br>Hapana 2<br>Sijui 96
d	Je, uliishi na mwanakaya ambaye alikuwa na msongo wa mawazo, mgonjwa wa akili au alikuwa na mawazo ya kujiua?	Ndiyo 1<br>Hapana 2<br>Sijui 96
e	Je, uliishi na mwanakaya ambaye alihukumiwa kwenda jela?	Ndiyo 1<br>Hapana 2<br>Sijui 96
f	Je, wazazi wako waliwahi kutengana au kutalikiana?	Ndiyo 1<br>Hapana 2<br>Sijui 96
g	Je, baba, mama au mlezi wako alifariki dunia?	Ndiyo 1<br>Hapana 2
h	Uliwahi kuona au kusikia mzazi au mtu mwingine nyumbani kwenu akipigwa kibao, akisukumwa au akipigwa ngumi?<br><br>Unaweza kusema haijawahi kutokea, mara moja au mara chache au mara nyingi?	Hata kidogo 1<br>Mara moja 2<br>Mara chache 3<br>Mara nyingi 4

		Hata kidogo	Mara moja	Mara chache	Mara nyingi
**700**	Maswali yafuatayo yanahusu baadhi ya vitu ambavyo inawezekana vilikupata WEWE MWENYEWE. Wakati ulipokuwa unakua, ndani ya kipindi cha miaka 18 ya mwanzo wa maisha yako; je, mzazi au mwanafamilia mwingine ambaye ni mtu mzima aliwahi…				
a	Kukuita majina mabaya, kukutukana au kukudharau?<br>Unaweza kusema haijawahi kutokea, mara moja au mara chache au mara nyingi.	1	2	3	4
b	Kutishia kukudhuru mwili<br>Je, haikutokea hata kidogo, au ilitokea mara moja, mara chache, au mara nyingi?	1	2	3	4
c	Kukupiga kofi matakoni au usoni, kukupiga teke, kukupiga ngumi au kukupiga sana tu?<br>Unaweza kusema haijawahi kutokea, mara moja au, mara chache au mara nyingi.	1	2	3	4
d	Kukupiga kwa nguvu sana mpaka ukapata majeraha au alama mwilini<br>Je, haikutokea hata kidogo, au ilitokea mara moja, mara chache, au mara nyingi?	1	2	3	4
e	Je, mtu mzima au anayekuzidi angalau miaka 5 alikugusa au kukutomasa kimapenzi?<br>Je, haikutokea hata kidogo, au ilitokea mara moja, mara chache, au mara nyingi?	1	2	3	4
f	Kukufanya wewe umtomase kimapenzi?<br>Je, haikutokea hata kidogo, au ilitokea mara moja, mara chache, au mara nyingi?	1	2	3	4

g	Kujaribu kufanya mapenzi na wewe kwa njia ya mdomo, haja kubwa, au ukeni?<br>Je, haikutokea hata kidogo, au ilitokea mara moja, mara chache, au mara nyingi?	1	2	3	4
h	Kwa hakika alifanya mapenzi na wewe kwa njia ya mdomo, haja kubwa, au ukeni?<br>Je, haikutokea hata kidogo, au ilitokea mara moja, mara chache, au mara nyingi?	1	2	3	4
**701**	Swali lifuatalo linahusu MAPIGANO YA KIMWILI. Mapigano ya kimwili hutokea wakati vijana wawili wenye nguvu na uwezo unaoelekea kulingana wanapoamua kupigana. Wakati unakua, kuanzia utotoni hadi kufikia umri wa miaka 18...	Hata kidogo	Mara moja	Mara chache	Mara nyingi
**702**	Ulifanya mapigano ya kimwili mara nyingi kiasi gani?	1	2	3	4
**703**	Wakati unakua, je, uliona ...<br>SOMA MAJIBU	Hata kidogo	Mara moja	Mara chache	Mara nyingi
a	Mtu katika jamii yako akipigwa?	1	2	3	4
b	Mtu akichomwa kisu?	1	2	3	4
c	Mtu akipigwa risasi?	1	2	3	4
d	Mtu akitishwa kwa bunduki au silaha nyingine?	1	2	3	4
e	Wezi au majambazi wakijaribu kuingia nyumbani kwenu?	1	2	3	4

SEHEMU YA 7: KUHUSU JAMII YAKO

Ningependa kukuuliza maswali kadhaa kuhusu maisha yako nje ya familia yako.

	MASWALI	AINA ZA MISIMBO	
701	Unapotoka kazini, unatumia muda wako mwingi ukiwa na nani?	Familia<br>Marafiki<br>Kanisani<br>Jamii<br>Kwingine, tafadhali taja ______________	1<br>2<br>3<br>4<br>5
702	Je, katika mwaka mmoja uliopita, umewahi kupigwa ngumi, mateke au kipigo kingine nje ya nyumbani?	Ndiyo<br>Hapana	1<br>2
703	Katika miezi 12 iliyopita, ni mara ngapi umefanya mapigano ya kimwili na watu wengine usioishi nao?	Hata kidogo<br>Mara moja<br>Mara chache<br>Mara nyingi	1<br>2<br>3<br>4
704	Je, umewahi kushiriki mapigano ya kutumia kisu, bunduki au silaha nyingine?	Hata kidogo<br>Mara moja<br>mara 2-3<br>Mara nyingi zaidi	1<br>2<br>3<br>4
705	Je, umewahi kukamatwa na polisi?	Ndiyo<br>Hapana	1<br>2

Sasa ningependa kujua zaidi kidogo kuhusu vikundi na vyama ulivyojiunga navyo katika jamii yako.

706-707	Tafadhali niambie iwapo umejiunga na kundi au makundi yafuatayo:	706		707<br>Kama **jibu ni NDIYO kwa swali namba 706,** tafadhali niambie pia iwapo huwa unahudhuria tu, au unashiriki kikamilifu au kama hata ni kiongozi wa baadhi ya shughuli za kikundi.		
		Ndiyo	Hapana	Mwanachama /anahudhuria	Hai	Kiongozi
a.	Kikundi cha kidini	1	2	1	2	3
b.	Vikundi vya kikabila/ Vikundi vya kitamaduni	1	2	1	2	3
c.	Vikundi vya msaada wa kiuchumi (visivyotoa mikopo)	1	2	1	2	3
d.	Vikundivyamtaa	1	2	1	2	3
e.	Vikundi vya sherehe/ na kuzikana	1	2	1	2	3
f.	Kikundi cha vijana	1	2	1	2	3
h.	Kamati ya shule	1	2	1	2	3
i.	Kamati ya afya	1	2	1	2	3
j.	Kikundi cha michezo	1	2	1	2	3
k.	Kikundi cha mikopo/kifedha	1	2	1	2	3
l.	Chama cha kisheria/ kitaalamu	1	2	1	2	3
m.	Vikundi vya utetezi	1	2	1	2	3
n.	Vingine (taja): ____________________	1	2	1	2	3

708	**Kama mtu/ shirika litatoa mafunzo kuhusu jinsi ya kuboresha mahusiano kati ya wanaume na wanawake...**<br>Je, utapenda kushiriki?	Ndiyo 1<br>Hapana 2	 Rukia swali la 710
709	Kama jibu ni ndiyo: sababu yako kuu ya kupenda kushiriki ni ipi?	Ninataka kuboresha 1<br>uhusiano 2<br>Ninapenda watoto 3<br>Ninataka kujiendeleza 4<br>Nyingineyo:<br>______________	Rukia swali la 711<br>Rukia swali la 711<br>Rukia swali la 711<br>Rukia swali la 711
710	Kama jibu ni hapana: sababu yako kuu ya kutopenda kushiriki ni ipi? Si muhimu, Nimetingwa na kazi, Sababu nyingine?	Si muhimu 1<br>Nimetingwa na kazi 2<br>Sina muda 3<br>Si kazi ya wanaume 4<br>Sababu nyingine: _________ 5	

Sasa nitakuuliza maswali kadhaa kuhusu jinsi jamii hii inavyoendesha mambo yake na inavyoshughulikia matatizo.

711	Majirani katika jamii hii wanaelekea kujuana vizuri	Ninakubaliana kabisa<br>Ninakubaliana<br>Sikubaliani<br>Sikubaliani kabisa	1<br>2<br>3<br>4
712	Katika jamii hii, kwa jumla watu huaminiana katika masuala ya kukopeshana na kuazimana.	Ninakubaliana kabisa<br>Ninakubaliana<br>Sikubaliani<br>Sikubaliani kabisa	1<br>2<br>3<br>4
713	Katika eneo hili, ni salama kutembea wakati wa usiku	Ninakubaliana kabisa<br>Ninakubaliana<br>Sikubaliani<br>Sikubaliani kabisa	1<br>2<br>3<br>4
714	Tuchukulie kwamba watu wawili katika kijiji/ kitongoji hiki wana mgogoro mkubwa baina yao. Kimsingi, unadhani ni nani atasaidia kusuluhisha mgogoro huo?	Hakuna mtu; watajisuluhisha wenyewe.<br>Wanafamilia/ wanakaya<br>Majirani<br>Wanavikundi<br>Viongozi wa jamii<br>Viongozi wa dini<br>Viongozi wa mahakama<br>Wengine (taja):	1<br><br>2<br>3<br>4<br>5<br>6<br>7<br>8
715	Unahisi kwamba unajiamini kiasi gani kuomba ushauri kutoka kwajirani yako au rafiki yako?Je, unaweza kusema kwamba: (SOMA MAJIBU)	Ninajiamini sana<br>Ninajiamini lakini ninahitaji kutiwa moyo<br>Sijiamini hata kidogo<br>Sijui	1<br>2<br>3<br>96
716	Mara nyingi majirani wana matatizo (kwa mfano, kuhusu malezi ya watoto, mahusiano au kupata kazi).Unajiamini una uwezo kiasi gani kutoa ushauri kwa jirani yako au rafiki yako? Je, unaweza kusema kwamba: (SOMA MAJIBU)	Ninajiamini sana<br>Ninajiamini lakini ninahitaji kutiwa moyo<br>Sijiamini hata kidogo<br>Sijui	1<br>2<br>3<br>96

BAADA YA KUMALIZA...

Maelezo Kuhusu Mahojiano - MWISHO

Tarehe ya mahojiano:

Muda wa kumalizika mahojiano:

Jina la mhojaji:

Je, wewe ni mhojaji yuleyule wa mwanzo? HapanaNdiyo

Maoni:

Kufunga mahojiano

Ninapenda kukushukuru sana kwa kutusaidia. Ninakushukuru kwa muda wako ulioutumia. Ninatambua kwamba pengine ilikuwa vigumu kujibu maswali haya,lakini njia pekee ambayo sisi tunaweza kuelewa vizuri masuala ya mahusiano na uzoefu katika maisha ni kwa kuwasikiliza nyinyi wanaume wenyewe mkiyaeleza.
