# Supplementary material for: Couples data from north-western Tanzania: Insights from a survey of male partners of women enrolled in the MAISHA cluster randomized trial of an intimate partner violence prevention intervention
Source: PLoS One. 2020 Oct 2;15(10):e0240112. doi: 10.1371/journal.pone.0240112 (PMC7531846; doi:10.1371/journal.pone.0240112)
Supplement: S1 Table — (DOCX) [file pone.0240112.s005.docx]

**S1 Table: Variables used in analysis of factors associated with IPV**

|  | Variable construction |
| --- | --- |
| **Outcome variables** |  |
| Past year physical IPV by current partner | Binary: Yes; No  Coded as ‘Yes’ if she reports that her current partner has done at least one of the following things to her in the past year:   - Slapped her or thrown something at her that could hurt her - Pushed her or shoved her or pulled her hair - Hit her with his fist or something else that could hurt her - Kicked her, dragged her or beat her up - Choked or burnt her on purpose - Threatened to use or actually used a gun, knife or other weapon against her   Coded as ‘No’ if these things haven’t been done to her in the past year, or if they have been done by a previous partner only. |
| Past year sexual IPV by current partner | Binary: Yes; No  Coded as ‘Yes’ if she reports that any of the following have happened to her in the past year:   - A husband/partner forced her to have sexual intercourse by threatening her, holding her down or hurting her in some way - She had sexual intercourse when she did not want to because she was afraid that her partner would hurt her or someone she cared about if she refused - She had sexual intercourse when she did not want to because she was afraid that her partner would leave her or take another girlfriend if she refused   Coded as ‘No’ if these things haven’t been done to her in the past year, or if they have been done by a previous partner only. |
| **Childhood factors** |  |
| Man witnessed violence against a household member as a child | Binary: Yes (once or more); No (never)  Respondent asked about the first 15 years of their life: “Did you see or hear a parent or household member in your home being slapped, kicked, punched or beaten with a fist or object?” (Response options: Never; Once; Few times; Many times) |
| Man experienced sexual abuse or severe physical abuse as a child | Binary: Yes; No  Coded as ‘Yes’ if respondent reports that a parent or other adult household member ever did at least one of the following things to them in the first 18 years of their life:   - Hit you so hard that you had marks or were injured   Or that a person at least 5 years older than them ever   - touched or fondled you in a sexual way? - made you touch their body in a sexual way? - attempted oral, anal or vaginal intercourse with you - actually had oral, anal or vaginal intercourse with you? |
| Man witnessed violence against a household member as a child, or experienced sexual abuse or severe physical abuse herself as a child | Binary: Yes; No  Coded as ‘Yes’ if experienced either sexual/severe physical abuse as a child, or witnessed violence against a household member as a child (as defined in the above two outcomes) |
| Man’s highest level of school completed | Binary: None/primary or below; Attended secondary or higher |
| Woman witnessed violence against a household member as a child | Binary: Yes (once or more); No (never)  Respondent asked about the first 15 years of their life: “Did you see or hear a parent or household member in your home being slapped, kicked, punched or beaten with a fist or object?” (Response options: Never; Once; Few times; Many times) |
| Woman experienced sexual abuse or severe physical abuse as a child | Binary: Yes; No  Coded as ‘Yes’ if respondent reports that a parent or other adult household member ever did at least one of the following things to them in the first 15 years of their life:   - Hit you so hard that you had marks or were injured   Or that a person at least 5 years older than them ever   - touched or fondled you in a sexual way? - made you touch their body in a sexual way? - attempted oral, anal or vaginal intercourse with you - actually had oral, anal or vaginal intercourse with you? |
| Woman witnessed violence against a household member as a child, or experienced sexual abuse or severe physical abuse herself as a child | Binary: Yes; No  Coded as ‘Yes’ if experienced either sexual/severe physical abuse as a child, or witnessed violence against a household member as a child (as defined in the above two outcomes) |
| Woman’s highest level of school completed | Binary: None/primary or below; Attended secondary or higher |
| **Current demographics** |  |
| Man’s age | Categorical: 19-39 years; 40-49 years; 50+ years |
| Man’s income | Categorical: Monthly quartiles (1=highest, 4=lowest); Doesn’t earn  Based on reported daily, weekly or monthly income in Tanzanian Shillings. Reported daily and weekly earnings were converted to monthly earnings on the assumption that each participant worked for 22 days per month. A higher quartile indicates higher income. |
| Woman’s age | Categorical: 19-39 years; 40-49 years; 50+ years |
| Woman’s income | Categorical: Monthly quartiles (1=highest, 4=lowest); Doesn’t earn; Don’t know  Based on reported daily, weekly or monthly income in Tanzanian Shillings. Reported daily and weekly earnings were converted to monthly earnings on the assumption that each participant worked for 22 days per month. A higher quartile indicates higher income. |
| Duration of relationship | Ordered categorical: 10+ years; 5-9.99 years; <5 years |
| Man has other wife/wives | Binary: One wife(partner); More than one wife (as reported by man) |
| Age-gap | Binary: Man >5yrs older; Same age (within 5 years)/woman older  Calculated from self-reported age of each |
| Relative education status | Categorical: Neither has above primary; just she does; just he does; both do  Based on self-reported highest level of education of each |
| Children living in household that have a different biological father to male respondent | Binary: Yes; No |
| Combined monthly income quartile | Categorical: Monthly quartiles (1=highest, 4=lowest); Don’t know  Calculated from combined total of self-reported income of man and woman |
| Either partner reported household-level financial hardship in past year | Categorical: Either/both partner(s) coded as reporting financial hardship; Neither reported hardship  Respondents were asked a series of questions:  During the last 12 months…   1. how many times were you very worried/stressed about your general financial situation? 2. have you had trouble buying food or other necessities for your family 3. have you had to borrow money to pay rent or other bills? 4. did any of your family members need to see a doctor but could not because you did not have enough money? 5. did your children miss days of school because you did not have money for school fees, uniform or supplies? 6. Have you or any of your children gone a whole day without eating anything because there was not enough food?   (Response options: Never; Once; Few times; Many times)  Respondents were coded as having experienced household-level hardship in the past year if they answered:   - ‘A few times’ or ‘Many times’ to (a)   *and*   - either:   - ‘A few times’ or ‘Many times’ to any of (b)-(f)   *or*   - - ‘Once’ to at least two of (b)-(f) |
| **Attitudes and health** |  |
| Man’s attitudes on IPV | Binary: Accepting of IPV; Not accepting of IPV  Coded as ‘Accepting’ if ‘Strongly agrees’ or ‘agrees’ that a man has good reason to hit his wife in at least one of the following scenarios:   - She does not complete her household work to his satisfaction - She disobeys him - She refuses to have sexual intercourse with him - She protests because he has other girlfriends - He suspects that she is unfaithful in marriage - He finds out that she has been unfaithful in marriage |
| Man’s poor mental health | Binary: Yes; No  Coded as ‘Yes’ if scored 8 or more in SRQ-20 |
| Man’s alcohol use | Categorical: Doesn’t drink; <2-3 times per week; 2-3 times or more per week |
| Woman’s attitudes on IPV | Binary: Accepting of IPV; Not accepting of IPV  Coded as ‘Accepting’ if ‘Strongly agrees’ or ‘agrees’ that a man has good reason to hit his wife in at least one of the following scenarios:   - She does not complete her household work to his satisfaction - She disobeys him - She refuses to have sexual intercourse with him - She protests because he has other girlfriends - He suspects that she is unfaithful in marriage - He finds out that she has been unfaithful in marriage |
| Woman’s poor mental health | Binary: Yes; No  Coded as ‘Yes’ if scored 8 or more in SRQ-20 |
| Woman’s alcohol use | Binary: Doesn’t drink alcohol; Drinks alcohol |
