# Supplementary material for: Couples data from north-western Tanzania: Insights from a survey of male partners of women enrolled in the MAISHA cluster randomized trial of an intimate partner violence prevention intervention
Source: PLoS One. 2020 Oct 2;15(10):e0240112. doi: 10.1371/journal.pone.0240112 (PMC7531846; doi:10.1371/journal.pone.0240112)
Supplement: S2 Table — (a) Odds ratios of association between women’s/relationship characteristics and women’s consent to invite partner for interview; (b) Odds ratios of association between women’s experience of IPV and women’s consent to invite partner for interview, disaggregated by trial arm. (DOCX) [file pone.0240112.s006.docx]

S2 Table: Odds ratios of association between women’s/relationship characteristics and women’s consent to invite partner for interview

|  | Woman consented | OR* (95%CI) |
| --- | --- | --- |
|  |  |  |
| Currently married/living as married |  |  |
| *No* | 43/148 (29%) | - |
| *Yes* | 469/642 (73%) | 6.62 (4.54 – 9.66) |
| Same partner as at baseline |  |  |
| *Yes* | 479/707 (68%) | - |
| *No* | 22/66 (33%) | 0.24 (0.13 – 0.42) |
| Male partner has other wife |  |  |
| *No* | 390/545 (72%) | - |
| *Yes* | 99/206 (48%) | 0.37 (0.28 – 0.49) |
| Age |  |  |
| *<30yrs* | 46/68 (68%) | - |
| *30-39yrs* | 197/295 (67%) | 0.96 (0.50 – 1.86) |
| *40-49yrs* | 177/291 (61%) | 0.74 (0.39 – 1.41) |
| *50+* | 81/119 (68%) | 1.02 (0.52 – 2.00) |
| Partner’s age |  |  |
| *<40yrs* | 121/185 (65%) | - |
| *40-49yrs* | 183/272 (67%) | 1.09 (0.72 – 1.63) |
| *50+* | 187/288 (65%) | 0.98 (0.65 – 1.47) |
| *Don’t know* | 2/5 (40%) | 0.35 (0.05 – 2.75) |
| Education |  |  |
| *None/incomplete primary* | 66/101 (65%) | - |
| *Completed primary* | 328/504 (65%) | 0.99 (0.66 – 1.47) |
| *Attended secondary or higher* | 107/168 (64%) | 0.93 (0.60 – 1.45) |
| Has at least one child |  |  |
| *Yes* | 482/737 (65%) | - |
| *No* | 30/53 (57%) | 0.69 (0.40 – 1.19) |
| Monthly income |  |  |
| *1^st^ quartile* | 123/190 (65%) | - |
| *2^nd^ quartile* | 103/166 (62%) | 0.89 (0.58 – 1.37) |
| *3^rd^ quartile* | 116/176 (66%) | 1.05 (0.66 – 1.68) |
| *4^th^ quartile* | 119/182 (65%) | 1.03 (0.69 – 1.53) |
| *Doesn’t earn* | 31/45 (69%) | 1.21 (0.57 – 2.53) |
| *Don’t know* | 20/31 (65%) | 0.99 (0.47 – 2.08) |
| Contributes more financially to household than partner does |  |  |
| *Less than/same as* | 334/479 (70%) | - |
| *More than* | 176/303 (58%) | 0.60 (0.43 – 0.83) |
| Currently borrowing from BRAC |  |  |
| *Yes* | 307/458 (67%) | - |
| *No* | 205/332 (62%) | 0.79 (0.58 – 1.10) |
| Experienced household financial hardship in past year |  |  |
| *No* | 220/342 (64%) | - |
| *Yes* | 292/448 (65%) | 1.04 (0.75 – 1.44) |
| Poor mental health |  |  |
| *No* | 306/482 (63%) | - |
| *Yes* | 206/308 (67%) | 1.16 (0.88 – 1.53) |
| Seen partner drunk many times in the past year |  |  |
| *Never* | 349/527 (66%) | - |
| *Once/a few times* | 74/115 (64%) | 0.92 (0.64 – 1.33) |
| *Many* | 88/144 (61%) | 0.80 (0.54 – 1.18) |
| Attitudes condoning IPV |  |  |
| *No* | 263/401 (66%) | - |
| *Yes* | 249/389 (64%) | 0.93 (0.71 – 1.23) |
| Good communication with partner (discussing their day/feelings) |  |  |
| *No* | 189/341 (55%) | - |
| *Yes* | 323/449 (72%) | 2.06 (1.55 – 2.75) |
| Very confident to assert an opinion if different to partner’s |  |  |
| *Not confident/would need encouragement* | 133/229 (58%) | - |
| *Very confident* | 379/561 (68%) | 1.50 (1.06 – 2.13) |

*Odds ratios calculated from logistic regression models, with cluster robust standard errors used to take account of the clustered nature of the data (loan group as the cluster variable).

Supplementary Table 6b: Odds ratios of association between women’s experience of IPV and women’s consent to invite partner for interview, disaggregated by trial arm

|  | Control | | Intervention | |  |
| --- | --- | --- | --- | --- | --- |
|  | Woman consented | OR (95%CI)* | Woman consented | OR (95%CI)* | Likelihood Ratio Test  p-value** |
| Past year experience of sexual and/or physical IPV |  |  |  |  |  |
| *No* | 166/257 (65%) | - | 194/301 (64%) | - |  |
| *Yes* | 64/118 (54%) | 0.65 (0.46 – 0.92) | 88/114 (77%) | 1.87 (1.11 – 3.14) | P=0.002 |
| Past year experience of physical IPV |  |  |  |  |  |
| *No* | 188/294 (64%) | - | 229/346 (66%) | - |  |
| *Yes* | 42/81 (52%) | 0.61 (0.38 – 0.97) | 53/69 (77%) | 1.69 (0.84 – 3.39) | P=0.009 |
| Past year experience of sexual IPV |  |  |  |  |  |
| *No* | 187/301 (62%) | - | 221/333 (66%) | - |  |
| *Yes* | 43/74 (58%) | 0.85 (0.53 – 1.36) | 61/82 (74%) | 1.47 (0.93 – 2.34) | P=0.146 |

*Odds ratios calculated from logistic regression models, fitted separately for intervention and control women, with cluster robust standard errors used to take account of the clustered nature of the data (loan group as the cluster variable).

**Likelihood ratio tests compared models with and without interaction terms between trial arm and IPV variable. In order to allow the LRT test, these models did not adjust for clustering.
