# Supplementary material for: Couples data from north-western Tanzania: Insights from a survey of male partners of women enrolled in the MAISHA cluster randomized trial of an intimate partner violence prevention intervention
Source: PLoS One. 2020 Oct 2;15(10):e0240112. doi: 10.1371/journal.pone.0240112 (PMC7531846; doi:10.1371/journal.pone.0240112)
Supplement: Attachment — Submitted filename: Plos bReview 24032020.docx [file pone.0240112.s007.docx]

**Factors associated with intimate partner violence in north-western Tanzania: Results from a survey of male partners of women enrolled in the MAISHA cluster randomised**

**trial**

Thank you for the opportunity to review this paper that is a valuable contribution to literature that focuses on the dynamics of conducting violence related research among couples. I have several comments to make which may be helpful to the authors

Title: Although the paper does look at factors associated with women’s victimisation and corroborates data from male partners, the current title misses the main objective and novelty of the paper which is the process and selection associated with women’s willingness to have male partners participating in research with them. It may be good to reflect this somehow in the title if possible.

Introduction: This is well written and has logical flow including a strong rationale for the study.

Methods:

It is clear that male partners were not asked about violence perpetration to minimise risks to female participants. However, in line 138 there’s reference that men were asked about relationship characteristics and dynamics. Male participants were also questioned about attitudes towards IPV. It will be important for authors to also discuss the potential for risk associated with administering such questions with male partners in context of them being abusive. It may be without asking about violence perpetration there is chance of suspicion by male partners who may well be guarded about their partners discussing their relationships with outsiders.

Results:

The authors report on insignificant associations i.e 95%CI overlapping with 1 as “weak associations”. This is problematic and must be rectified including discussions that allude to these as follows:

Line 320 Physical IPV is associated with financial hardship

Line 324 men’s attitudes are said to be weakly associated with physical and sexual IPV

Line 327 -typo- 95% CI from table is 1.03-3.08

Line 328- Men’s alcohol is associated with physical IPV

Line 331- There is “suggestion “that women’s alcohol is weakly associated with physical IPV

Overall authors must use the standard that every OR with 95%CI overlapping with 1 should not be considered as a significant weak association.

Discussion:

The discussion must be revised in accordance to changes in interpretation of results as alluded above.

The finding that men’s violence perpetration was not associated with their attitudes towards IPV warrants further discussion. Could that have been a result of social desirability biases or how else can this be explained.

The discussion does not provide explanation about what could be happening with women who did not disclose IPV but still were unwilling for their male partners to participate. These may be an important group to understand

Limitations

A missed opportunity is that researchers did not explore women’s motivations for their un/willingness to let their male partners participate in the study. It is important to cite this as a limitation. However, the speculated explanations based on data distribution of women who consented is well argued.
